# Molecular epidemiology of *Salmonella* Enteritidis in humans and animals in Spain

**DOI:** 10.1128/aac.00738-24

**Published:** 2025-03-03

**Authors:** Clara Samper-Cativiela, Laura Torre-Fuentes, Bernabé Diéguez-Roda, Margo Maex, María Ugarte-Ruiz, Paula Carrizo, Marta Hernández, Úrsula Höfle, José Luis Sáez, Cristina de Frutos, Montserrat Agüero, Miguel Ángel Moreno, Lucas Domínguez, Silvia Herrera-León, Julio Alvarez

**Affiliations:** 1 VISAVET Health Surveillance Centre, Complutense University of Madrid 16734 , Madrid, Spain; 2 Department of Animal Health, Faculty of Veterinary Medicine, Complutense University of Madrid 16734 , Madrid, Spain; 3 TRAGSATEC, Tecnologías y Servicios Agrarios S.A. 707474 , Madrid, Spain; 4 Division of Human Bacterial Diseases, Sciensano, Uccle, Belgium; 5 Departamento de Anatomía Patológica, Microbiología, Medicina Preventiva y Salud Pública, Medicina Legal y Forense. Facultad de Medicina, Universidad de Valladolid Facultad de Medicina 88194 , Valladolid, Spain; 6 IREC, Instituto de Investigación en Recursos Cinegéticos 69555 , Ciudad Real, Spain; 7 Subdirección General de Sanidad e Higiene Animal y Trazabilidad, Dirección General de la Producción Agraria, Ministerio de Agricultura, Pesca y Alimentación, Madrid, Spain; 8 Laboratorio Central de Veterinaria, Ministerio de Agricultura, Pesca y Alimentación, Algete, Spain; 9 Laboratorio de Referencia e Investigación en Enfermedades Bacterianas Transmitidas por Alimentos, Instituto de Salud Carlos III 38176 , Madrid, Spain; Johns Hopkins University School of Medicine, Baltimore, Maryland, USA

**Keywords:** *Salmonella*, molecular epidemiology, foodborne zoonosis, whole genome sequencing, antimicrobial resistance, one health

## Abstract

*Salmonella* Enteritidis, the most prevalent serovar-causing human gastroenteritis, has been traditionally linked to poultry sources. Although antimicrobial resistance (AMR) is not common in this serovar, increasing levels of resistance to fluoroquinolones and ampicillin have been reported in the last few years. Here, 298 isolates retrieved from different sources (human, livestock, wildlife, food, and environment) and years (2002–2021) in Spain were analyzed to evaluate their diversity, the distribution of AMR-conferring genes (ARGs), and mutations and reconstruct the epidemiology of infection due to this serovar. Isolates were clustered in two major clades (I and II), with strains in clade I (including 61.5% of all human isolates) displaying a pan-susceptible phenotype and not carrying AMR determinants. In contrast, clade II included 80.7% of isolates from animal/food/environmental sources, with the majority (69.8%) harboring mutations in the quinolone resistance determinant regions (QRDR). ARGs, although rare, were mostly found in clade II strains that also carried plasmid replicons, among which IncX1 was the most common. Although higher levels of phenotypic resistance were found in animal isolates, extended-spectrum beta-lactamase, plasmid-mediated AmpC, and carbapenemase-encoding genes were only found among human isolates. In summary, the majority of human and animal isolates from Spanish sources in our collection were classified in different phylogenetic branches, suggesting that additional sources are contributing to the occurrence of foodborne infections in Spain. Furthermore, the different distributions of virulence factors and ARGs in isolates from different sources and their association with specific plasmids suggest the presence of different dynamics contributing to the selection of resistant strains.

## INTRODUCTION

Foodborne salmonellosis is the second most common enteric bacterial disease reported in Europe and North America, with over 60,050 human cases reported in 2021 in the European Union (EU) (1, 2). Different non-typhoidal *Salmonella* serovars may act as human pathogens, but *Salmonella enterica* subsp. *enterica* serovar Enteritidis (hereafter referred to as *S*. Enteritidis) has been the most commonly reported serovar in the majority of industrialized countries in the last decades (3). In Europe, over 54.6% of all notified cases reported in 2021 as well as a large proportion (79.7%) of the reported foodborne outbreaks were caused by this serovar (2).

*S*. Enteritidis is considered a generalist *Salmonella* serovar, which means that it does not have a strong preference for a given host and can infect a broad range of animal species (4, 5). However, shelled eggs and poultry meat are typically considered the main vehicles of transmission to humans (6, 7). The success of industrialized poultry farming from the 1970s onward has been linked to the rise in *S*. Enteritidis-related human cases, possibly due to the improvement in biosecurity measures targeting other poultry-adapted serovars (*Salmonella* Gallinarum and *Salmonella* Pullorum), which opened an ecological niche for the former to become established in poultry flocks (8, 9). Since the 1990s, this serovar has surpassed *Salmonella* Typhiumurium as the most commonly associated with human cases worldwide (10). Nowadays, *S*. Enteritidis is the main target (along with *S*. Typhimurium and its monophasic variant) of the strategies for monitoring and control of *Salmonella* along the different stages of the poultry production chain (farms, processing plants, retail, and wholesale) (11–13).

*S*. Enteritidis frequently induces mild self-limiting gastroenteritis, but when invasive infection occurs, therapy may be needed (14). In such cases, the presence of resistant isolates, in particular multidrug-resistant (MDR, defined as resistant to three or more antimicrobial classes) isolates, can complicate the treatment (15–17). Fortunately, and in contrast to what is observed in other serovars, *S*. Enteritidis is typically susceptible to most antimicrobials: around 72.1% of the isolates recovered from human cases notified in European member states (MSs) in 2021 were susceptible to all antimicrobials tested. Nevertheless, several European countries have reported increasing trends in antimicrobial resistance (AMR) in clinical *S*. Enteritidis isolates over the 2013–2021 period (18); in 2021, the proportion of isolates resistant to certain antimicrobial classes classified as highest priority critical importance antibiotics (CIA) (19) reached 22.6% (fluoroquinolones) and 3.4% (beta-lactams) (18). High levels of colistin resistance have also been reported, a phenomenon that had been previously attributed to the natural tolerance to this antibiotic in *S*. Enteritidis due to the lipopolysaccharide (LPS) membrane composition observed in serovars belonging to group D (20). However, a mutation in the promoter region of the *mgrB* gene, a regulator of the PhoP/PhoQ system involved in colistin resistance, present exclusively in *S*. Enteritidis ST11 isolates has been recently associated with colistin resistance in this serovar (21).

Several *S*. Enteritidis multicounty outbreaks involving up to several hundreds of cases have occurred in the last decades both in Europe and internationally (22, 23). In some cases, as in the well-characterized polyclonal outbreak related to Polish eggs first detected in 2016, these outbreaks may last several years (11, 24), further highlighting the complex epidemiology of the infection due to this serovar. In this context, the increasing application of whole-genome sequencing (WGS) allows more reliable identification of outbreak sources (24–26) and is gradually replacing traditional typing methods in epidemiological surveillance (27, 28). Its use also allows the identification of *S*. Enteritidis characteristic AMR and virulence determinants (28), as well as the assessment of the possible role of specific transmission mechanisms (e.g., plasmids and other mobilizable elements) in their dispersion (29). Several studies have also proven the usefulness of WGS to detect the presence of predominant *S*. Enteritidis lineages both locally and worldwide and track their evolution (9, 30, 31). Despite the increase in the use of WGS worldwide, this technique is still not routinely applied in certain major poultry-producing countries like Spain, resulting in its underrepresentation in international studies focused on the global dynamics and lineage distributions of this serovar (9, 23, 31–33). Here, we performed a WGS-based study on a large panel of *S*. Enteritidis isolates retrieved from a wide range of sources encompassing humans, livestock, wildlife, and food in the last 16 years to identify the presence of major clades, infer the level of bacterial exchange between host populations, and determine the main genetic mechanisms conferring resistance to antimicrobials with a focus on CIA classes and mobile genetic elements (MGE) involved in horizontal gene transfer (HGT).

## RESULTS

Overall, 298 isolates from humans (*n* = 122), broiler (including broiler meat, *n* = 54), laying hen (*n* = 44), eggs (*n* = 50), and other sources (livestock, wildlife, environmental; *n* = 28) retrieved over the 2002–2021 period were included in our study. *In silico* serotyping and MLST typing confirmed the identity of the 298 sequenced isolates as *S*. Enteritidis belonging to sequence types (ST) ST11 (*n* = 295), ST3233 (*n* = 2, retrieved from turkey and broiler breeder), and ST183 (*n* = 1, from wild boar) (Data Set S1).

### AMR determinants and MGE

Besides the *aac(6’)-Iaa* cryptic gene, found in all isolates and not considered from here on, up to 26 different AMR determinants were detected in 185 of 298 sequenced isolates, including 22 ARGs (present in 45 isolates) and four different chromosomal point mutations in the *gyr*A gene (present in 157 isolates). These AMR determinants conferred resistance to eight different antimicrobial classes (aminoglycosides, beta-lactams, phenicols, lincosamides, quinolones/fluoroquinolones, sulphonamides, tetracyclines, and trimethoprim) and antiseptics (quaternary ammonium compounds) (Data Set S1; Table S1). The most common antimicrobial class involved in the genetic and phenotypical resistance detected was quinolones, followed by colistin and beta-lactams (Table 1).

**TABLE 1:** Agreement between the observed phenotypes and predicted resistance determinants per tested antimicrobials

Resistance determinants	Antimicrobial	Genotype-phenotype^*a*^				K [95% CI]^*b*^	*P*	N^*c*^
		G+/P+	G+/P-	G-/P+	G-/P-			
*gyr*A [87:D-N, D-Y]; [83:S-F, S-Y]	Quinolones (NAL)	126	7	5	157	0.92 [0.85–0.98]	<0.0005	295
*gyr*A [87:D-N, D-Y]; [83:S-F, S-Y]*; qnr*S1*; qnr*B19	Fluoroquinolones (CIP)	100	29	4	158	0.77 [0.66–0.87]	<0.0005	291
*bla*_TEM-1b_; *bla*_TEM-1d_; *bla*_CMY-2_; *bla*_SHV-12_; *bla*_CTX-M-14_; *bla*_CTX-M-15_; *bla*_TEM-52C_; *bla*_OXA-48_	Beta-lactams (AMP)	31	5	3	252	0.87 [0.79–0.95]	<0.0005	291
*tet*(A)*; tet*(B)*; tet*(K)	Tetracyclines (TET)	6	2	3	280	0.70 [0.58–0.81]	<0.0005	291
*sul*1*, sul*3	Sulphonamides (SMX)	5	1	13	245	0.4 [0.24–0.55]	<0.0005	264
*dfr*A1	Trimethoprim (TMP)	4	0	1	230	0.89 [0.80–0.97]	<0.0005	235
*cml*A1	Chloramphenicol (CHL)	2	0	11	272	0.26 [0.10–0.42]	<0.0005	285
mgrB (A :: T)	Polimyxin (CST)	97	30	6	31	0.49 [0.3–0.7]	<0.0005	164

^*a*^ G+: resistance determinant detected; G-: resistance determinant not detected; P+: resistance phenotype; P-: susceptible phenotype.

^*b*^ Kappa agreement and 95% CI.

^*c*^ N : total number of isolates with information on their phenotypic profile per antimicrobial.

The observed and predicted (based on the presence of AMR determinants) resistance phenotype was evaluated separately for eight antibiotics, tested among different numbers of isolates ranging from 164 (colistin) to 295 (nalidixic acid) (Tables 1 and 2). The agreement was high (kappa = 0.81–1) for nalidixic acid, beta-lactams, and trimethoprim; moderate (kappa≈0.70) for ciprofloxacin and tetracycline; and limited (kappa ≤40) for polymyxins (colistin), sulphonamides, and chloramphenicol (Table 1). The A/T substitution in the *mgrB* gene promoter previously linked to colistin-resistance was present in 117 of the 298 isolates of our collection but was only partially associated with the resistance phenotype: 97 of the 103 colistin-resistant isolates had the T genotype, but this genotype was also found in almost half (30/61) of the colistin-susceptible isolates.

**TABLE 2:** Antimicrobial resistance determinants in the isolate collection (*n* = 298)

No. of determinants	Determinant pattern	Human P1^*a*^	Human P2^*b*^	Human outbreaks	Human surveillance (sporadic)	Broiler related^*c*^	Laying hen related^*c*^	Eggs	Other	Total
Total isolates		**29**	**14**	**22**	**57**	**54**	**44**	**50**	**28**	**298**
0	Pansusceptible	25	1	17	35	12	19	11	14	134
1	Total 1 determinant	4	6	5	22	38	17	38	13	143
	gyrA p. D87N	1			1		1		1	4
	gyrA p. D87Y	2	2	2	10	36	15	32	11	110
	gyrA p. S83F				1	1				2
	gyrA p. S83Y			3						3
	*bla*_TEM-1B_				10	1	1	6		18
	*bla*_CMY-2_		1							1
	*bla*_OXA-48_		1							1
	*bla*_TEM-52C_		2							2
	*qnr*B19	1								1
	*tet*(K)								1	1
2	Total 2 determinants		3			2	4	1	1	11
	*gyr*A *p*. D87Y*, bla*_CMY-2_					1				1
	*gyr*A *p*. D87Y*, bla*_CTX-M-14_		1							1
	*gyr*A *p.* D87Y*, bla*_SHV-12_		2							2
	*gyr*A *p*. D87Y*, bla*_TEM-1D_						4			4
	*gyr*A *p.* D87Y*, tet*(B)								1	1
	*gyr*A *p. D*87Y*, qac*J					1				1
	*bla_TEM-1B_, lnu(A)*							1		1
3	Total 3 determinants		1			2				3
	*gyr*A *p.* D87Y*, bla*_TEM1D_*, bla*_CTX-M-15_^*d*^		1							1
	*gyr*A *p.* D87Y*, bla*_TEM1B_ *, qnr*S1^*d*^					1				1
	***gyr*A *p.* D87Y*, lnu*(A)*, tet*(K)^*e*^**					**1**				**1**
**4**	**Total 4 determinants^*e*^**		**1**				**3**			**4**
	***gyr*A *p.* D83F*, aad*A1*, bla*_SHV-12_*, qac*E^*e*^**		**1**							**1**
	***aad*A1*, sul*1*, tet*(A)*, dfr*A1^*e*^**						**3**			**3**
**5**	**Total 5 determinants^*e*^**						**1**			**1**
	***gyrA p. S83F, aadA1, sul1, tet(A), dfrA1^e^***						**1**			**1**
***7***	**Total 7 determinants^*e*^**		**2**							**2**
	***gyrAp.D87Y,aadA1,aadA2b,*** ***bla*_*SHV-12*_ *, cmlA1, sul3, tet(A)^e^***		**2**							**2**
Total patterns	**24 different patterns**	**3**	**9**	**2**	**4**	**7**	**7**	**3**	**4**	**24**

^*a*^ Isolates sequenced as part of a project to compare invasive vs. non-invasive infections.

^*b*^ Isolates sequenced as part of a project focused on resistant strains.

^*c*^ Includes isolates from fecal or meat samples.

^*d*^ Three AMR determinants found but resistance to less than three antimicrobial families.

^*e*^ MDR isolates (resistance to more than three antibiotic families) are shown in bold.

The most prevalent AMR determinants were chromosomal point mutations in the *gyr*A quinolone resistance determinant region (QRDR), present in 136 of the 298 isolates. Four different aminoacidic changes in two different *gyr*A codons (34, 35) were found: D87Y was present in 125 isolates; D87N and S83F were present in four isolates each; and S83Y was found in three isolates. In addition, two isolates carried a plasmid-mediated quinolone resistance (PMQR) gene (*qnr*S1, present in a broiler isolate with a D87Y mutation with elevated MICs to both CIP and NAL, and *qnr*B19, in a human isolate with no mutations in the QRDRs and susceptible to NAL and CIP) (Table 2).

The remaining 20 ARGs (other than *qnr* genes) were found in a low proportion of isolates (14.7%, 44/298). Among these, beta-lactam-associated ARGs were the most common (Table 2; Table S1), with up to eight different beta-lactamase encoding genes detected in a total of 36 isolates. Extended-spectrum beta-lactamase (ESBL) encoding genes were found in nine isolates (which originated from human samples), including *bla*_SHV-12_ (five isolates), *bla*_TEM-52C_ (two isolates), and *bla*_CTX-M-14_ and *bla*_CTX-M-15_ (one isolate each). The *bla*_CMY-2_ plasmid-mediated AmpC- b-lactamase (pAmpC) gene was present in one human and one broiler isolate, whereas the carbapenemase-encoding gene *bla*_OXA-48_ was present in one human isolate. In addition, two *bla*_TEM-1_ variants were detected in 25 isolates: *bla*_TEM-1B_ was present in 10 isolates from humans, seven from eggs, and one from broiler breeder, broilers, and laying hens, and *bla*_TEM-1D_ was found in four isolates from laying hens and one from a human case (Table 2).

Regarding other antimicrobial classes, nine isolates harbored tetracycline-related genes (*tet*[A], *tet*[B], and *tet*[K]), seven isolates harbored aminoglycoside-resistant genes (*aad*A1 in all seven and *aad*A2b in two), six genes conferring resistance to sulfamethoxazole (*su1*1 or *sul*3)*,* four to trimethoprim (*dfr*A1), two isolates carried genes conferring resistance to lincosamides (*lnu*[A]), and two isolates two different antiseptics/efflux pump genes (*qac*E and *qac*J) (Table 2).

Many isolates presenting AMR determinants typically harbored only one (143 isolates), although 11 isolates presented two, and 10 carried ≥3 determinants. Overall, eight of these 10 isolates were classified as multidrug-resistant based on their genotype (i.e., carrying determinants conferring resistance to ≥3 antimicrobial classes) (Table 2). Of these, four were retrieved from laying hens, three from human cases, and one from broiler (Table 2).

Thirteen different plasmid replicons linked with both short (<7,000 pb) and long (>50,000 pb) size plasmids were identified in the isolate collection. Both the IncFIB and IncFII replicons associated with the 60 kb virulence plasmid pSEV were found in 281/298 isolates. Among the remaining 17 isolates, one (the single ST183 strain) carried IncFII only, the other carried FIB only, and the other 15 did not carry either. The remaining replicons belonged to five incompatibility (Inc) groups (IncX1, IncX3, IncX4, IncI1-I[Alpha], and IncL), present in between one and 36 isolates each, to three rep plasmid groups (rep13, rep21, and rep7a) present in one to three isolates, and to five Col plasmid groups (ColRNA, Col156, ColpVC, Col[pHAD28], and Col440I) present in two to 30 isolates. When considering plasmid groups other than IncFIB and IncFII, replicons were present, single or in combination, in a total of 58 isolates (1). Up to 71% of the isolates presenting these replicons (*n* = 41) also carried from one to six ARGs (and thus a plasmid replicon was not detected in only 4/45 isolates carrying at least one ARG). The most prevalent plasmid replicon (other than IncFIB and IncFII) was IncX1, present in 36 isolates (of which 26 harbored at least one ARG), followed by IncI1[Alpha], present in 11 isolates (seven isolates also harbored ARGs). Two other Inc replicons (IncX3 and IncL), present in one isolate each, were detected in isolates carrying one ARG (*bla*_SHV-12_ and *bla*_OXA-48_, respectively). Finally, two of the three isolates carrying rep7a, one of the two isolates harboring rep21, and the two isolates with rep13 also harbored ARGs. None of the 30 isolates carrying ColRNA plasmids carried ARGs, but 29 originated from human cases (and 26 were clustered in clade ID, see below).

### Plasmid characterization

The preliminary analysis of the genetic context (based on short reads assemblies and MOB-suite extracted sequences) in isolates carrying both replicons and ARGs indicated that the latter were typically contained in the putative plasmid sequences (i.e., in the same contig where the plasmid replicon was found) (Table S2): IncX1 replicons were associated with a variety of genes including *aad*A1, *bla*_TEM-1B_, *bla*_TEM-1D*,*_ *dfr*A1*_,_ qnr*S1*, sul*1*, tet(*A*),* and *tet(*B*)*; IncX3 replicon with *bla*_SHV-12_; IncL with *bla*_OXA-48_; IncI1-I(Alpha) with *aad*A1, *aad*A2b, *bla*_SHV-1*2*_, *bla*_CMY-2_, *bla*_CTX-M-14_, *cml*A1, *qac*E, *sul*3, and *tet(*A*),* rep13 with *lnu*(A); rep7a with *tet(*K*)* and rep 21 with *qac*J (Table S2).

**Fig 1:**
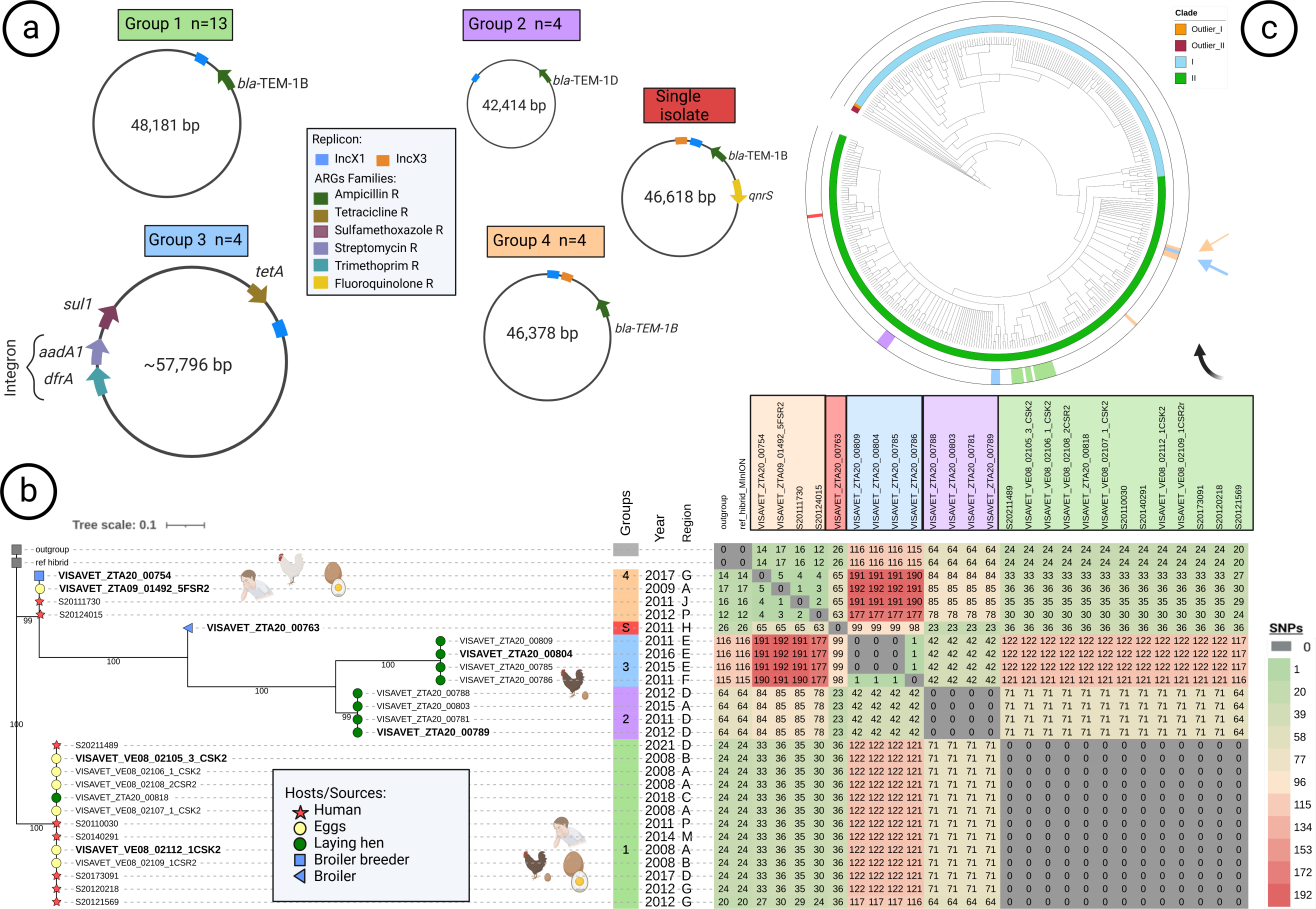
Combined representation of plasmid composition (size, replicon, and ARGs content) and phylogenetic relationship of 26 plasmid sequences presenting ARGs in IncX1(and IncX1-IncX3) plasmids. (**a**) Five different IncX1 (and IncX3) plasmid sequence structures showing size, replicon, and ARGs approximate location and ARGs family resistance. (**b**) Maximum-likelihood phylogeny based on SNPs presence after alignment with a hybrid reference (see methodology) of these 26 plasmid sequences, where symbol branches represent host/source of the samples, and the color strip shows the plasmid group of classification. Here, year and region of isolation are shown, and SNPs figures matrix is presented on the right side. (**c**) Global phylogeny of 338 isolates showing clades and location of isolates presenting IncX1 plasmid sequence by the color of the plasmid group. Arrows showed an area where group 3 and group 4 plasmids were located.

After the visualization of hybrid assemblies (and MOB-suite detected in such cases) and with support from the phylogenetic analysis of the 26 IncX1 ARGs-related sequences, we obtained a classification of these plasmid sequences into four groups (three of the original 29 sequences with ARGs were removed due to low quality), and one of the 26 was classified alone, here named single isolate (Fig. 1a and b). The maximum SNP difference between plasmid sequences allocated in the same group was five (Fig. 1b). The relaxase type of all these plasmid sequences was MOBP, classified as conjugative (Table S2).

The most prevalent group (Group 1), present in 13/26 isolates coming from seven different regions, contained the *bla*_TEM-1B_ gene and had a sequence of 48,180 bp long. Group one plasmids were found in six isolates retrieved from eggs (all from 2008), one from laying hen (from 2018), and six from human samples (from 2011 to 2021) (Fig. 1a). Plasmids belonging to Group 2 (42,414 bp long) contained the *bla*_TEM-1D_ gene and were found in four isolates from laying hen from two Spanish regions retrieved in different years (Fig. 1a). Plasmid sequences within both Group 1 and Group 2 were identical. Plasmids classified in Group 3 had the longest sequence (57,796 bp long) and contained the *aadA1*, *sul*1, *tet(*A*)*, and *dfr*A1 ARGs (*aad*A1 and *dfr*A1 as part of a class one integron) and were found in four isolates from laying hen retrieved from two regions in different years over the 2011–2016 period. Their sequences only differed by one SNP (Fig. 1a). Finally, the plasmids conforming Group 4 (46,378 bp long and harboring *bla*_TEM-1B_) were found in four isolates, two coming from clinical cases in consecutive years (2011–2012) but different regions, one isolate from eggs and one from broiler breeders retrieved in 2009 and 2017 in two different regions, with plasmid sequences differing by five SNPs; further annotation of their sequences revealed that presented both IncX1 and IncX3 replicons. The broiler breeder isolates presented also co-integration of the *bla*_TEM-1D_ gene in IncFIB/IncFII. The remaining IncX1 plasmid sequence (presenting also IncX1 and IncX3 replicons) was not ascribed to any group (single isolate) and contained the *bla*_TEM-1B_ and *qnr*S1 quinolone resistance gene (Fig. 1a and b).

Only two plasmid sequences containing the IncI1-(Alpha) replicon and ARGs were correctly characterized as being part of a plasmid by the bioinformatic tool MOB-suite and were compared with another for which hybrid assembly was available. These two (92,422 bp long) were identical (100% coverage and identity) (Table S2) carried the *bla*_CMY-2_ gene and were found in isolates retrieved from broiler (2009) and human (2021) samples. The remaining plasmid sequence (114,757 bp) carried six ARGs (*aad*A1*, aad*A2, and *cml*A1 in a class 1 integron, plus *bla*_SHV-12_*, sul*3 and *tet*[A]) and was carried by a human isolate retrieved in 2005. No plasmid groups were identified among the plasmid sequences belonging to the remaining replicon types. All rep plasmids, present in isolates from animal sources, presented MOBV relaxase type (Table S2).

### Phylogenetic analysis

**Fig 2:**
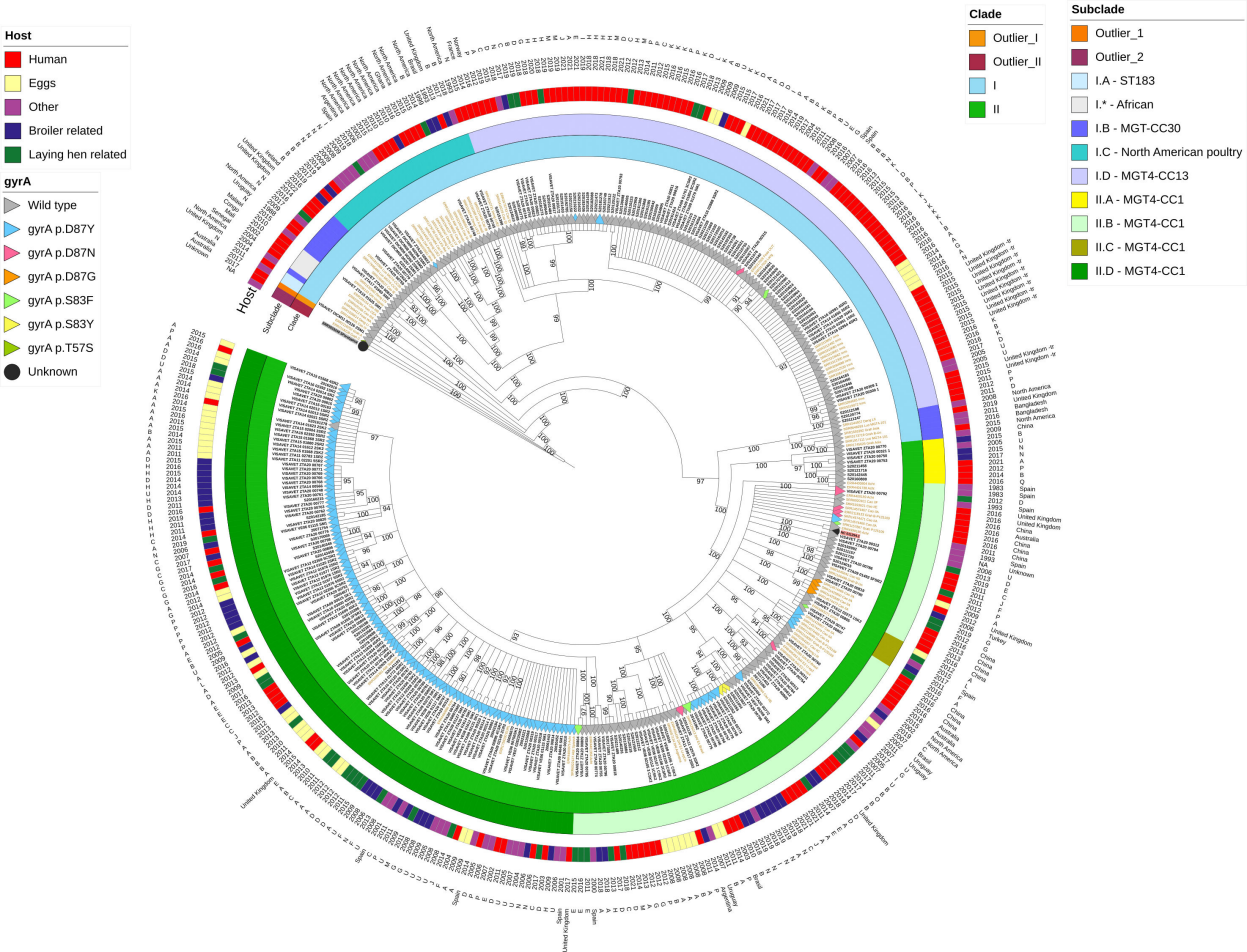
Phylogenetic relationship of 388 S. Enteritidis isolates. Dendrogram representation (circular) of maximum-likelihood phylogeny based on SNP presence (P125109 reference label showed in pink, S. Parathypi outgroup in gray). Isolates from other studies are labeled in gold and isolates from this study in black. Branch symbol shows *gyrA* mutation types).

The 298 sequenced isolates differed by 0–1,669SNPs. When the hierBAPS (hierarchical Bayesian Analysis of Population Structure) algorithm was applied to the 388 isolates included in the phylogeny, these were clustered into four BAPS clades (outlier I, outlier II, clade I, and clade II). Two of these clades (outliers I and II) only included *S*. Enteritidis isolates from other studies (36), and the *S*. ParathyphiA strain used as outgroup (>1,700 SNPs), and thus, they were considered outlier clades here (Fig. 2). The two main clades I and II were then subdivided into five subclades (IA to ID and I*) and four subclades (IIA to IID), respectively.

Considering only the sequences from this study (298), clade I contained 109 isolates (36.6%) that were more genetically heterogeneous (median SNP distance = 94, Interquartile Range (IQR) = 51–158) compared with the remaining 189 isolates (63.4%) included in clade II (median SNP distance = 68, IQR = 47–86) (Fig. 2). The median between-clade SNP distance was 416 SNPs (IQR = 404–427). Among the 109 isolates of clade I, 75 isolates (68.1%) came from human sources, 12.8% from the broiler and laying hen-related categories, 7.3% from eggs, and 11.0% from other sources (including environmental and wildlife) (Fig. 2). In contrast, 44.5% of the clade II isolates originated from broiler and laying-hen related sources, 22.2% from eggs, 8.5% from environmental/wildlife, and only 24.9% were retrieved from human samples. Human isolates classified in clade I included primarily those related to foodborne outbreaks (*n* = 18/22) and sporadic cases (55/88, project Human P1 and surveillance), whereas clade II encompassed 12/14 human isolates selected due to their resistance phenotype to third-generation cephalosporins (project Human P2). Overall, 61.5% of the 122 human isolates included in our isolates collection were included in clade I, whereas 85.1% of the 148 isolates from eggs and broiler and laying hen-related sources belonged to clade II. Of the 28 isolates categorized as coming from other sources (mostly environmental/wildlife) a slightly higher percentage was also grouped in clade II (57.1%) (Fig. 2).

Once the two outlier clades were excluded, the 86 remaining external isolates (i.e., those that were sequenced in the frame of other studies) were allocated in similar proportions in the two major clades, 48 in clade I and 39 in clade II (Fig. 2). Considering all 388 isolates used in the phylogenetic analysis, 157 isolates were allocated in clade I (Fig. 2). The predominant subclade here (ID) included 68.8% of the clade I isolates with a high proportion of the Spanish human isolates from our study collection (~70%) and 15 external isolates; 12 of these 15 were also isolates from human cases from a Spanish travel-associated outbreak (25). Subclades IA, IB, and IC were made up of a large proportion of external isolates and only contained one, three, and 12 isolates from our collection respectively, whereas clade I* included only external strains (Fig. 2) (32, 37). Within the four subclades of clade II, IIB, and IID were the most represented ones (41.7% and 52.6% clade II isolates, respectively), with 64.5% and 81.7% of their isolates originating from food/animal/environmental sources each. Although clade IIB included a high proportion of external isolates (37) from several studies and various origins (Europe, America, and Asia), all external isolates classified in clade IID (*n* = 5) had been retrieved in Spain or were linked to travel to the country (38). Subclades IIA and IIC only contained eight and five isolates, respectively, and all isolates in IIC lacked the IncFIB and IncFII replicons. All major clades and subclades included isolates from different decades. When the same analysis was carried out including only isolates from the most sampled timeframe within the study period including all sources (2011–2019, 241 isolates, Fig. S1), a similar picture was observed with the same major clades identified accounting for similar proportions of isolates from the different sources (Fig. S2).

**Fig 3:**
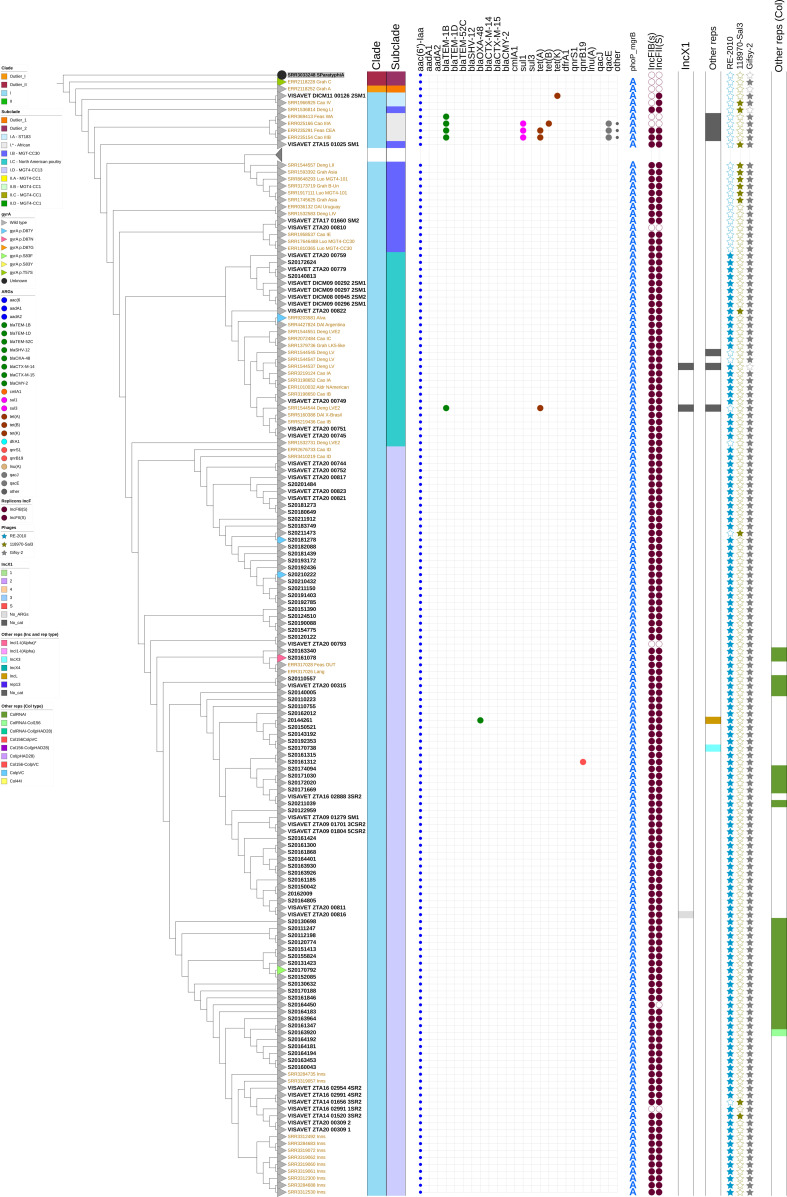

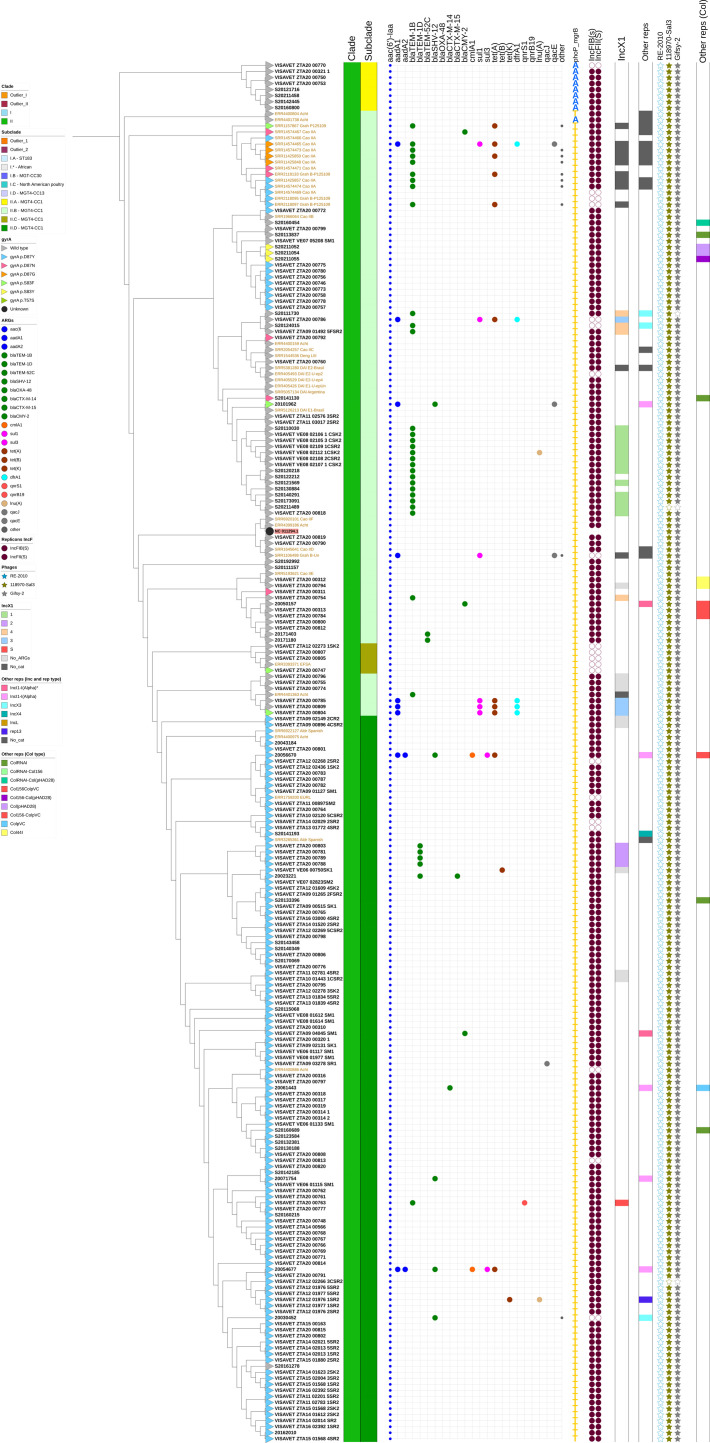
Phylogenetic relationship of 388 *S*. Enteritidis isolates. Top: Clade I and outlier clade dendrogram (rectangular) showing in addition to traits described in Fig 2 (clade or branch symbols showing *gryA* mutation types) the presence of ARGs, phoP_mgrB point mutation, plasmid replicons IncF, IncX1, other Inc replicons, phages and other Col type replicons. Bottom: Clade II dendrogram showing same traits as stated above

In terms of antimicrobial resistance, 102 of the 109 isolates from our collection in clade I (93.5%) did not present any AMR determinant, with the remaining isolates carrying either a mutation in the QRDR regions (*n* = 4) or another ARG (one of *bla*_OXA-48_, *qnr*B19 or *tet*[K]) (Fig. 3; Table 2). In contrast, 83.1% (157/189) of the isolates from our collection in clade II contained one or more AMR determinants, with a majority (69.8%, 132/189) harboring at least one mutation in the QRDR (Fig. 3; Table 2). Among *gyr*A mutations, D87Y was the most represented one in this clade (123/189), followed by D87N, and S83F and S83T with only three isolates each. However, although 72.5% of the isolates in subclade IIB presented a wildtype QRDR genotype, all the isolates in clade IID contained the same *gyr*A mutation (D87Y) (Fig. 3). All human isolates selected based on their resistance phenotype (Human P2), which carried ESBL/pAmpC genes were allocated in subclade IID, whereas the only one presenting resistance to carbapenems was allocated in clade I (Fig. 3). The A/T mutation of the *mgrB* promoter was also strongly associated to the BAPS clade, with all 109 isolates in clade I presenting the wild-type genotype and 181/189 isolates in clade II harboring the mutated genotype (all but the eight isolates in subclade IIA) (Fig. 3).

All isolates carrying ARGs in IncX1 plasmids were placed in clade II (Fig. 1c and 3). Isolates carrying plasmids from the IncX1-G1 and IncX1-G2 groups were clustered together in the phylogeny, with <45 SNPs between their genomes (Fig. 1c and 3). In contrast, one isolate carrying plasmids in the IncX1-G3 and in the IncX1-G4 groups was located in different branches from the remaining isolates of these same groups (with at least 75 and 84 SNP differences, respectively) (Fig. 1c and 3).

Based on the SNP address analysis up to 29 5-SNP clusters containing between two and 11 isolates (for a total of 92 isolates) were detected in the full collection including external isolates. Twenty-two of these clusters belonged to clade II (IIB, IIC, and IID), and the remaining seven were classified in clade I (ID) (Fig. S3). Four of the 29 clusters contained more than five isolates (from six to eleven) and were all located in clade II, and the remaining 25 clusters contained two to five isolates. The largest 5-SNPs cluster (*n* = 11) included 10 isolates from broiler (one of them from broiler meat) and one from humans retrieved from 2011 to 2019 in three different regions (with three broiler isolates from 2011, 2014, and 2015 and the human isolate from 2016 originating from the same region) (Fig. S3). The second largest 5-SNPs (*n* = 8) cluster contained seven isolates from broiler and one from turkey retrieved in a short period (2018–2019) but from five different Spanish regions. Two other 5-SNP clusters containing seven and six isolates each included exclusively isolates from eggs. The remaining 18 5-SNP clusters in clade II, containing between two and five isolates, included 1–2 human isolates along with isolates from non-human sources (eggs, broiler, or laying hens) in four of them, with human and non-human isolates retrieved in the same or the following year in three clusters. All 5-SNPs clusters located in clade I were only formed by pairs of human isolates (Fig. S3).

### Clade-specific prophages, SPIs, virulence factors, and accessory genes

The most prevalent prophage, present in 381/388 isolates and duplicated in four isolates, was Gifsy 2 (NC_010393), followed by 118970-sal3 (NC_031940), present in 239 isolates, and RE-2010 (NC_019488) in 130 isolates (Fig. 3). These last two prophages were only present simultaneously in two isolates (VISAVET_ZTA14_01520_3SR2, VISAVET_ZTA20_00822). RE-2010 was mainly found in isolates belonging to clade I (subclades IC and ID), whereas prophage 118970-sal3 was generally present in isolates from clade II and observed only in five isolates from clade I (subclades IB, IC, ID) (Fig. 3).

A total of 41 *Salmonella* pathogenicity islands (SPIs) were detected in the 388 isolates (S2 Fig), 10 of which were present in all the isolates and contained genes involved in functions such as iron uptake system, type III secretion system, transcriptional regulation Mg2^+^ uptake (*Mgt*C, B, *Mar*T, and *Mis*L), intestinal colonization, and persistence determinants (*shd*A, *rat*A, *rat*B, *siv*I, and *siv*H). Another 19 islands were present in between 360 and 387 isolates, whereas the remaining 12 SPIs were only found in between 1 and 20 isolates (Fig. S4).

No particular association between the presence of virulence genes and the BAPS clade was found: a total of 183 virulence genes were identified across the 388 isolates (Fig. S2), 96 of which were present in all isolates with another 46 only missing in between one and 36 isolates. Of note, the *sspH2* gene, involved in the colonization of host cells by *S*. Enteritidis, was missing in 32 isolates from clade ID (26 of which originated from humans) (Fig. S4, Data Set S1). The remaining 41 virulence genes were present in between one and 24 isolates.

The pangenome analysis identified 13 gene sequences whose presence was linked with subclade I and 58 associated with clade II (Table S3). According to their functional annotation, genes present exclusively or predominantly in clade II were linked to metabolic and regulatory processes including carbohydrate metabolism (PTS_EIIA_2), stress response mechanisms (*gcdB*), as well as nutrient transport and signal transduction pathways, supporting their role in environmental adaptation and survival. In addition, several gene sequences linked to both clades placed contiguously in the genomes were associated with phages further supporting the differential distribution of phages in the different clades.

The pangenome analysis identified the presence of gene *dgcJ*, previously linked to bacterial motility (39–41), virulence, and immune system response (42–45), only in clade ID isolates. A BLAST analysis revealed that in fact, its sequence was truncated in ID isolates due to a 58 bp deletion, whereas the full sequence was present in the remaining clade I and all clade II isolates.

## DISCUSSION

*S*. Enteritidis emerged globally in the last decades of the 20th century (9, 46) and remains the serovar most frequently reported from human cases in the EU (47) and North America (9, 23). Poultry products (mainly eggs) are commonly reported as vehicles of this serovar (6), but its ability to adapt to different hosts and transmission niches has been previously demonstrated (5, 13). Although *S*. Enteritidis is considered one of the most genetically homogeneous serovars of *Salmonella* (48), different *S*. Enteritidis lineages that present unique genomic features (including AMR determinants), different geographical distributions (including international spread), and specific host associations have been reported in several studies (23, 25, 31–33, 36, 49).

In the present study, we studied a panel of *S*. Enteritidis isolates retrieved from different sources in Spain over a 17-year period. The predominance of ST11 among the isolates in our collection (295/298) is in agreement with previous studies considering human strains with a European (50) or international (31, 51) scope. The closely related ST3233 (differentiated from ST11 by one mutation in the *dna*N housekeeping gene) was found in two studied isolates classified in subclade IB (<100 SNP distance to other ST11 strains), indicating a close relation between these two STs as reported before (52). The remaining strain, originating from a wild boar, belonged to the ST183 and was classified in a subclade (IA) that only contained another ST183 external isolate retrieved from hedgehogs (53).

According to the phylogenetic analyses performed, two major clades (I and II) contained most of the isolates in our collection (Fig. 2). These two clades (with the exception of subclade IA) would be allocated in the previously named clade B (50), a classification supported also by other studies (33, 36, 49, 50) (Fig. 2). The most remarkable genomic difference between the two major clades was the distribution of AMR determinants and associated resistance phenotypes (Fig. 3). In agreement with current knowledge indicating that *S*. Enteritidis isolates are typically susceptible to most antimicrobials (18, 54), we found 134/298 isolates that did not harbor any AMR determinant (except for the presence of the cryptic aminoglycoside acetyltransferase gene *aac(6’)-Iaa*). However, 94% isolates in clade I did not carry resistance determinants compared with only 17% of the isolates in clade II. According to our analysis, clade I was more heterogeneous, compared with clade II in spite of including almost two-thirds of the isolates sequenced here (particularly those from animal sources originating from multiple regions in the country). The within-clade genetic variability is in line with findings in other articles (33), reporting that the mutation rate of MGT4-CC13 (equivalent to clade I here based on the location of the external strains included in the analysis) was significantly faster (1.5 times) than that of MGT4-CC1, which would correspond to clade II (49).

Subclade ID contained 93/109 (85.3%) of the isolates from this study included in clade I; isolates from our collection in this subclade were predominantly retrieved from humans (78.5%, 73/93), with only 16/93 (17.2%) isolates from poultry sources (laying hen, broiler, eggs, and meat) and four from other sources. Subclade ID isolates included external genomes from the so-called Atlantic clade (9) or MGT14-CC13 (33), a lineage previously reported to harbor less antimicrobial resistance determinants compared with the global epidemic clade MGT4-CC1 (9, 36, 49) in agreement with our findings. Divergence of MGT14-CC13 has been dated around 1946, around the beginning of modern poultry breeding on both sides of the Atlantic (9). Isolates from this lineage have been retrieved from several sources in North America and Europe, including poultry and clinical isolates part of large-scale outbreaks in Europe (24, 25, 55–57). Human isolates from our study in ID included most of the outbreak-related human isolates included in our collection (18/22 encompassing 18 different outbreaks from 2018 to 2021) along with isolates from an outbreak in United Kingdom suspected to be related to traveling to Spain (25); in addition, it also 53/86 (61.6%) of the isolates from sporadic cases analyzed here (categories “Human P1” and “Surveillance” in supplemental Data Set S1) and retrieved during the 2011–2021 period. In contrast, the low representation of isolates from poultry sources found in this subclade suggests that *S*. Enteritidis strains causing a significant proportion of human infections in Spain (as outbreaks or sporadic cases) during at least the 2011–2021 period would have likely originated from sources other than poultry production in the country, since these strains were not commonly found in food animals/food products over the same period. Nevertheless, these findings should be interpreted with care, given the possible biases impacting the representativeness of the human isolates included in our collection (selected among those submitted to the National Centre of Microbiology and that thus could have originated from more severe cases) in comparison with the animal isolates (collected through active random sampling in healthy animals and thus more likely to capture the true distribution of *S*. Enteritidis genotypes present in livestock).

Subclade IC, which accounted for 11.0% of clade I isolates from this study, included isolates from wild boar and several poultry productions grouped with strains previously classified as part of the North American poultry-associated clade (36, 38) or US lineage (9). This indicates that this US lineage/North American poultry-associated clade (38), predominant in poultry production in North America from 1969 to 2010 (23) and that has been also reported in poultry in the African continent (38), can be found in Spain yet likely at a lower prevalence.

The only isolates from pig and bovine included in the study were grouped in subclade IB (2.8% of clade I isolates from this study) along with one strain retrieved from laying hen and UK isolates from clinical cases belonging to the European clade MGT3-CC30 (49), thus indicating that they were not genetically related with those identified in the classic *S*. Enteritidis hosts (i.e., poultry) or clinical cases in Spain and would originate from sources containing international yet underrepresented lineages in Spain (23, 36, 49).

Finally, within clade I, four external human-invasive *S* Enteritidis strains classified before in Western and Central East African clades (32) (Cao et al.) formed the clearly different (> 774 SNPs) subclade I*, in which no Spanish isolates were found. This demonstrates a lack of relatedness of Spanish strains with this emerging lineage in sub-Saharan Africa. Nevertheless, considering the recent emergence of new *Salmonella* clones linked with travelers in poultry production in Europe (such as *Salmonella* Kentucky ST198) (58), some of which may pose a potential public health threat, their distribution should be monitored in the future as suggested before (59).

Isolates in clade II were associated with strains retrieved from multiple locations (Europe, Oceania, Asia, Africa, and North and South America) belonging to the so-called global epidemic clade (9) or MGT4-CC1, with a MRCA dated around 1975 (33). These external isolates were grouped in subclade IIB with 62 Spanish isolates (32.8% of the 189 clade II isolates from this study) coming from poultry (laying hen, broiler, and eggs) sources and (predominantly sporadic) human cases, with isolates from Spain typically forming separate clusters from those retrieved from other countries (Fig. 2). Point mutations associated with quinolone resistance was not common in this subclade (45/62 had a wild type genotype), but in contrast, 19/20 isolates carrying the *bla*_TEM-1B_ gene in IncX1 plasmids were allocated in this subclade.

Nevertheless, the majority of isolates sequenced in this study classified in clade II were allocated in subclade IID (60.8%), with the unique five external isolates included in this subclade originating also from Spain or being related to travel to this country (38) (Fig. 2). Aggregation of strains in “national/regional” lineages deriving from the global epidemic lineage has been also reported in *S*. Enteritidis from other countries including China (37), Chile, and Brazil (9). This “national” subclade was strongly associated with the presence of the D87Y QRDR point mutation (all but one isolate presenting it) and included primarily isolates from broiler, laying hens, and poultry products (eggs and meat) (83/115) with fewer isolates coming from human cases (21/115), suggesting an apparently reduced exposure of humans in Spain to isolates found in animals and food in this clade. Nineteen of the 21 human isolates (eight selected based on their resistance phenotype belonging to category “Human P2” and 13 from sporadic cases) were resistant to quinolones as expected based on their genotype (two isolates were susceptible in spite of carrying a mutation in the QRDR), indicating a strong association between quinolone-resistance and belonging to this subclade.

The predominance of beta-lactamase genes (subclade IIB) and QRDR mutations (subclade IID) as the “branding” resistance determinants among the isolates in this study is in line with the results from the EU AMR monitoring programs, which also identified these two resistance traits as the most common ones (albeit at a low frequency) in *S*. Enteritidis isolates (over 22.6% and 3.4% of resistant isolates to fluoroquinolone and beta-lactams in 2021) (18), and with studies including isolates from other regions of the world (9, 36, 49).

The D87Y (Asp87Tyr) mutation that was found in all but one of the isolates in the “national” subclade IID was also the predominant trait found in *S*. Enteritidis isolates resistant to nalidixic acid retrieved from a region in Spain (Asturias) in which an increase in the resistance levels to this antimicrobial had been registered between 2000 and 2008–2014 (60, 61). In previous studies, the high frequency of this mutation was interpreted as a sign of a clonal expansion (60, 62), a hypothesis that would agree with the results of the phylogenetic analysis conducted here, although isolates carrying the mutation were found throughout the study period (i.e., since 2002 in the case of human isolates and 2006 for poultry isolates). Furthermore, when considering only poultry isolates (laying hen, broiler eggs, or meat) an almost overall 2:1 ratio of strains carrying the D87Y mutation vs. the wild-type genotype (91 vs. 54) was observed throughout the 2006–2019 period during which these animal isolates were available (with wild-type isolates falling mostly into subclades ID or IIB), indicating a sustained circulation at a higher frequency of the variant harboring the mutation. However, this ratio was more balanced when considering separately laying hens (19 D87Y vs. 23 wild type) compared with broilers (40 vs. 13) or eggs (32 vs. 18).

The high levels of fluoroquinolone resistance found among isolates in clade IIB, widely distributed in food animals according to our results, is concerning since this antimicrobial class is among the first-line treatment options in certain cases of salmonellosis (19). The predominance of this lineage carrying resistance determinants in animal sources in Spain during the study period (especially in broiler and egg sources) could be associated with an increased selective pressure favoring these phenotypes in food animals as previously stated (63, 64), due to, for example, the use of enrofloxacin in certain poultry productions such as broiler and turkey but not in others (laying hen, in which fluoroquinolone-resistant strains were as mentioned not so widespread based on our results) (65). The withdrawal of its use in poultry in the United States in 2005 (66) could explain the predominance of the North American poultry clade (lacking QRDR mutations) in this country, although the increase in the proportion of ciprofloxacin-resistant *S*. Enteritidis retrieved from chicken, turkey, and associated food products reported by NARMS since 2017 (67) could indicate the expansion of the global epidemic clade through trade as previously speculated (38). In addition, we found mutations in the *gyrA* gene in 45% of the 20 isolates coming from wildlife (all in clade II and linked in all cases but one to the *gyr*A D87Y mutation), thus demonstrating the circulation of this resistance in this environmental niche (68).

Phenotypic resistance to ampicillin was linked to the presence of beta-lactamase encoding genes (*bla*_TEM-1B_ and *bla*_TEM-1D_ genes) present in IncX1 plasmids, which were only found in a subset of clade II isolates (19/20 isolates carrying *bla*_TEM-1B_ in subclade IIB and the remaining *bla*_TEM-1B_ isolate and all five *bla*_TEM-1D_ positive isolates in subclade IID) from animal, food, and human samples. The detailed analysis of the sequence of these plasmids revealed the presence of different plasmid types (groups) differing in length (from 42,414 to 48,180 bp) and sequence identity (<70% between groups) (Fig. 1). The ARG content (*bla*_TEM-1B_) and length (48 kb) of plasmids in Group 1, the most abundant, was similar to what was described for a plasmid found in 22 *S*. Enteritidis isolates retrieved between 2008 and 2014 from human cases in Asturias but that was absent in cases from 2000 (60, 61). Isolates carrying this plasmid originated mostly from eggs and humans and were all located close in the phylogenetic tree (between 1 and 45 SNP differences) (Fig. 1) but were retrieved over a 14-year span (2008 to 2021) and from multiple different regions, and therefore, it is unlikely that they were all epidemiologically related, although some were part of the same 5-SNP clusters, and thus, in some cases, epidemiologically related isolates could have been detected in different samples. Plasmids from Groups 2 (linked to *bla*_TEM-1B_ and in clade IIB isolates) and 4 (carrying *bla*_TEM-1D_ and in clade IID isolates) could be related to other plasmids described in clinical isolates from the same region that were more abundant in 2000 compared with 2008–2014 (60, 61), although here, they were all found in isolates retrieved between 2009 and 2017. Altogether these findings suggest that at least some variants of these plasmids have been circulating in *S*. Enteritidis in poultry and poultry products in multiple regions from Spain for over 10 years, occasionally causing human disease. The link between clade II and carriage of ARGs in IncX1 plasmids found here was also reported for isolates belonging to the global epidemic clade (36, 69). Plasmid groups associated with MDR phenotypes due to carriage of *aad*A1, *sul*1, *tet*(A), and *dfr*A1 genes identified here exclusively in isolates from laying hen should be subjected to particular attention due to their potential for AMR dissemination.

Interestingly, 10 of the 11 isolates carrying ESBL/pAmpC genes (*bla_SHV-12_, bla_TEM-52C_, bla_CTX-M-14_*, and *bla_CMY-2_*) sequenced here were retrieved from human cases, and all of them were allocated in clade II (subclades IIB and IID) (Fig. 3). Although these isolates were sequenced precisely due to their resistance to third generation cephalosporins and are therefore not representative of the strains causing infections in Spain, they were all epidemiologically independent and thus could be considered a representative sample of the multidrug-resistant clinical strains circulating in the country. Our results would then suggest that MDR clinical isolates carrying ESBL/pAmpC genes in Spain belong to the global epidemic clade, although these ARGs are not common in the animal reservoir. The only strain carrying a carbapenemase-encoding gene (the *bla*_OXA-48_ gene) was retrieved from a patient admitted to a hospital, thus confirming the rare circulation of these genes in *Salmonella*. Given the frequency of nosocomial infection with carbapenemase-producing enterobacteria (especially *Citrobacter*, *Escherichia coli,* and *Klebsiella*), the *Salmonella* strain may have acquired the gene through horizontal transfer within the hospital/patient (70, 71).

In addition, we have identified clusters of closely related strains based on previous strategies to define epidemiologically related isolates (5-SNPs clusters) (72) retrieved from different sources including in some cases isolates from livestock, food, and humans in the same cluster, which could indicate the persistence of certain strains along the food chain and thus a risk for the consumer. These results should be interpreted with care since variations in the methodology (e.g., different variant calling tools or quality thresholds for SNP-calling) could lead to different classifications of isolates into 5-SNPs clusters, highlighting the need for a standardized system for SNP-calling.

Although the 20 wildlife isolates sequenced here were distributed in both clades (eight and 12 in clades I and II, respectively), a difference depending on the host species/clade was observed: 5/8 clade I (subclade IB) isolates were retrieved from wild boar, whereas 11/12 clade II (subclades IIA, IIB, and IID) isolates were cultured from wild bird samples (1). These results indicate that different wild animals may have been exposed to different *S*. Enteritidis lineages, a fact that could be expected in the case of urban scavenger birds (storks) but not in other wild birds studied here, such as kestrel. A well-designed sampling would be nevertheless required in order to evaluate the prevalence of infection by different *S*. Enteritidis lineages in these host species.

In this study, we have proven the efficacy of WGS applied to isolates retrieved mostly through surveillance programs to provide an overall idea of the genetic diversity of *S*. Enteritidis circulating in humans, livestock, food, and wildlife sources in Spain. In summary, our results demonstrate that the two most prevalent lineages described worldwide (Atlantic-US and global epidemic) (5, 23, 32, 33) are present in the different sources sampled in Spain, with Spanish isolates mostly grouped in three subclades (ID, IIB, and IID accounting for 90.6% of the 298 isolates sequenced) two of which (ID and IIB) also included external strains from different sources in other countries, whereas IID was only formed by isolates linked to Spain. Furthermore, there was a strong association between the subclade and the presence of resistance determinants in the chromosome (QRDR mutations, found in all but one IID isolates compared with 27% of the IIB and only 4% of ID isolates) or in plasmids (found in <15% of the full collection but mostly beta-lactamase genes in IncX1 plasmids present only in subclade IIB – *bla*_TEM-1B_ – and/or IID –*bla*_TEM-1D_). Although there was a relative overlap between sources across these predominant subclades, human isolates were associated with the pansusceptible subclade ID (60% of human isolates versus 18% and 17% in subclades IIB and IID, respectively), whereas poultry isolates were more commonly part of the fluoroquinolone-resistant subclade IID (especially broiler and egg isolates, with ≥60% of isolates from these sources in this subclade and 43% of isolates from laying hen compared with 18%–32% in subclade IIB and 3%–16% in subclade ID from the three sources). Although sampling periods for each source were not identical (with older human isolates retrieved in 2002 versus 2006 for broilers, 2008 for eggs, and 2011 for laying hens), the consistency in the predominance of each subclade in human versus poultry across the study period reveals that the apparently limited overlap between the phylogenetic group in human versus poultry isolates has been stable in the last 15 years. This would agree with the limited resistance to ciprofloxacin/quinolones among human Spanish *S*. Enteritidis isolates in the last two decades according to EFSA (with in fact a decreasing trend in resistance during the 2013–2022 period) (18). Nevertheless, the reason for this limited overlap is unclear: although a large proportion of the human isolates sequenced here were selected randomly among those causing outbreaks and sporadic cases and could thus be considered representative of the strains causing foodborne salmonellosis due to *S*. Enteritidis in Spain, only isolates submitted to the National Centre of Microbiology were available to us, and thus, there is a chance that this may not include the full extent of infections (due to the inclusion of, e.g., isolates leading to outbreaks and/or more severe cases, which would be more likely to visit a hospital and have a sample collected). Furthermore, there could be a geographical bias in the human collection (with certain regions submitting more isolates than others) that could not be formally considered here. Nevertheless, the identification of certain genetic differences specific to clade IID isolates (and especially the truncation of the sequence of the gene *dgcJ*, previously linked to pathogenicity/virulence) could indicate a competitive advantage under certain conditions leading to a higher chance of being included in our collection, although further studies should be conducted in order to confirm this hypothesis. Furthermore, additional studies considering potential sources neglected in this study (such as, e.g., imported foods or non-reported foreign travel, recently highlighted as more important than previously thought) (73) would be needed in order to further clarify the epidemiology of *S*. Enteritidis infection in Spain, whose complexity this study has demonstrated.

## MATERIALS AND METHODS

### Study population

The study population was formed by 298 isolates coming from livestock, food, wildlife, and environmental and human sources retrieved between 2002 and 2021 (80.9% of all isolates recovered after 2011) (Fig. S1) and originating from all Autonomous Regions in Spain, although 38.2% of all isolates originated from Central Spain (Madrid, Castilla y León, and Castilla la Mancha) (Fig. S5) (1). A total of 95 isolates from domestic animals were selected from a collection of *Salmonella* isolates assembled in the frame of the official AMR surveillance program during 2004–2020 in Spain containing ~500 *S*. Enteritidis isolates. The selection of this set of isolates, originating from laying hen (46), broiler (37), broiler breeders (13), laying hen breeder (1), turkey (3), bovine (1), and swine (1) was based on the host, year, and AMR profiles (see below). In addition, 58 isolates from food sources (50 isolates from retail eggs and eight isolates from broiler meat) retrieved through a Spanish regional *Salmonella* surveillance program between 2008 and 2016 were also included. Moreover, 23 *S*. Enteritidis isolates from wildlife and environmental sources were incorporated: 20 isolates retrieved from wildlife through different research projects (14 from wild birds—seven from stork samples, two from kestrel, and one each from bustard, duck, eagle owl, cattle egret, and heron samples—five from wild boars, and one from a raccoon) along with three isolates retrieved from a sewage water treatment plant in the Madrid region.

Finally, WGS data from 122 clinical isolates from human cases selected from those submitted to the National Centre of Microbiology of the Instituto de Salud Carlos III between 2002 and 2021 in Spain and sequenced on an Illumina NextSeq platform (see below) were included. Of these, 29 isolates (Human P1) were sequenced in the frame of a study that assessed differences between sporadic cases of invasive and diarrhea-causing *Salmonella* (collected from 2013 to 2016, Human P1), 14 isolates (Human P2) were sporadic cases selected due to a resistance phenotype to third-generation cephalosporins (isolates from 2002 to 2017, with 10 retrieved between 2002 and 2010; Human P2), 22 isolates were included in the representation of 19 outbreaks that took place from 2018 to 2021 in Spain (with three isolates from a single outbreak included), and the remaining 62 isolates were sequenced as part of the routine surveillance activities on isolates from sporadic cases between 2011 and 2021 (Data Set S1). Of note, all human isolates except the 10 selected due to their resistance phenotype (part of project Human P2) were retrieved after 2010 (Fig. S6).

To represent the worldwide diversity of *S*. Enteritidis, WGS raw reads from 88 isolates were downloaded from the European Nucleotide Archive (ENA) and included in the phylogenetic analysis (see below). This selection comprised nine isolates included in previous studies from South America (Argentina, Brazil, and Uruguay), 17 from North America (United States of America), five from Oceania (Australia), 14 from Asia (China and Bangladesh), five from Africa (Malawi, Mali, Senegal, Ghana, and Congo), and 26 isolates from countries other than Spain in Europe (France, Norway, Ireland, Turkey, and United Kingdom, with 11 isolates suspected to be travel-associated cases linked to Spain). Finally, 12 Spanish isolates available in Enterobase for which information on the isolation year was available were also included (Data Set S1).

Information on the antimicrobial susceptibility profile of all livestock, wildlife, environmental, and food isolates was determined using the 2-fold broth microdilution method (ISO 20776-1:2021). Seven antimicrobials (ampicillin, ciprofloxacin, chloramphenicol, gentamicin, nalidixic acid, tetracycline, and trimethoprim) were tested in 166 of the 176 isolates from non-human sources, with results for between one and four additional antimicrobials (colistin, meropenem, sulfamethoxazole, and tigecycline) available for subsets of isolates (Data Set S1). Minimum inhibitory concentrations (MICs) were dichotomized into wild-type (here referred to as “sensitive”) or non-wild-type (referred to as “resistant”) categories using the epidemiological cutoffs (ECOFFs) indicated by the European Committee on Antimicrobial Susceptibility Testing (EUCAST) (Table S4).

All human isolates were tested using the disk diffusion (DD) method, and the results were interpreted using the EUCAST or (when not available) the Clinical Laboratory Standards Institute (CLSI) clinical breakpoint guidelines (Table S4). Results for the same seven antibiotics mentioned above were available in the 122 human isolates, and additional antibiotics (one to four, same as above) were also available for subsets of isolates (Data Set S1). Finally, cefotaxime, amoxicillin + clavulanic, ceftazidime, and cefepime were tested in between eight and 14 human isolates retrieved as part of the same project (Human P2) (Data Set S1).

Resistance profiles were associated with the origin of the isolate: A large proportion of the animal and egg isolates were phenotypically resistant to fluoroquinolones (and other antimicrobials in some cases) reflecting the predominant phenotypes in the strain collections from which these originated while human isolates were predominantly susceptible (i.e., <20% resistance to any antimicrobial) except for those selected due to their resistance phenotype (Human P2) (Fig. S7).

### Bacterial DNA extraction and genome sequencing

All livestock, food, wildlife, and environmental isolates (*n* = 176) were sequenced in an Illumina MiSeq platform. DNA was extracted and purified using commercial kits (74), and paired-end libraries were prepared by the standard protocols. All isolates from human clinical cases (122) were sequenced in an Illumina NextSeq platform. DNA was extracted and paired-end libraries were prepared using commercial kits (see Appendix S1 for details).

A set of 13 isolates (see in results), representing a diversity of plasmid replicons of interest containing ARGs, were selected for long-read sequencing on two different MinION Nanopore platforms (Oxford Nanopore Technologies (ONT), UK). DNA extraction, long-read libraries, and base-calling were performed differently depending on the institution (74, 75) (Data Set S1) (see Text S1 in the supplemental material for more details).

### Genome assembly and global characterization

Illumina reads were trimmed with Trimmomatic (76) and evaluated with FastQC (77) (version and parameters used for these and other software tools are shown in Table S5). Then, reads that passed the quality control were assembled using SPAdes (78), and the quality of the assemblies was evaluated with QUAST (79), so that contigs below 200 base pair (bp) long were discarded. The serotype was verified using the *Salmonella In Silico* Typing Resource (SISTR) (80), assemblies were annotated using Prokka (81), and Multilocus sequence typing (MLST) was performed with the mlst software (T. Seemann, https://github.com/tseemann/mlst). Assemblies were screened for the presence of point mutations associated with AMR using PointFinder (82), and of antibiotic resistance genes, plasmid replicons, and virulence genes using ABRicate (https://github.com/tseemann/abricate) against different databases: ResFinder (83, 84), PlasmidFinder (34), and Virulence Factors Database (VFDB) (85), respectively. The presence of prophages was investigated with PHAST (PHAge Search Tool) and PhastER (86, 87).

Agreement between the predicted (presence of AMR-related genes/chromosomal mutations) and observed resistance phenotype was determined for each antimicrobial using two by two tables and quantified using Cohen’s kappa test through the “caret” package in R (Table 1) (35, 88).

Putative plasmid sequences were identified and extracted from the assembled genomes using MOB-suite (89). Isolates in which MOB-suite identified sequences with outlier plasmid lengths, inconclusive cluster IDs, and/or multiple contigs as part of a single plasmid were not considered reliable and thus discarded (Table S3). In the isolates harboring both plasmid replicons and AMR genes (ARGs), the genetic context (chromosomal or plasmid) of the ARGs (Data Set S1) was determined by characterizing the presumptive plasmid sequences identified in terms of ARGs and integron content using again ResFinder and IntegronFinder (90). ARGs-carrying plasmid sequences from the most prevalent incompatibility group (IncX1, see results) were preliminary clustered in putative plasmid groups based on sequence similarity (>99.5% identity and >99% coverage) of plasmid-containing contigs using BLAST (91) (Table S2). The remaining putative plasmid sequences presenting other plasmid replicons (IncI1-I(Alpha), rep7a, rep13, rep21, IncX3, IncL) were also compared using BLAST (Table S3).

For the 13 isolates selected for ONT long-read sequencing (see above), the FullForcePlasmidAssembler (FFPA) tool (92) was the genome assembly pipeline used for hybrid genome assembly, combining long (ONT) and short (Illumina), as previously described (75). In this pipeline, quality of long reads was checked with NanoStat and NanoPlot (93), interspecies contamination are assessed by Kraken (94). Adapter and barcode sequences were removed with Qcat (https://github.com/nanoporetech/qcat/tree/master/qcat), and low-quality (<q8) reads were removed with NanoFilt (93). Complete genome sequences were obtained using Unicycler (95). FFPA tool uses ABRicate (https://github.com/tseemann/abricate) to scan the genomes for ARGs (ResFinder database) and plasmid replicons (PlasmidFinder database) identification.

Once the genomes were assembled and the assembly graphs visualized using Bandage (96), plasmid and chromosome sequences were examined separately, and further analyses for confirmation of the Illumina-based BLAST IncX1 plasmid group classification were conducted (Tables S3 and S6). Thus, a phylogeny based on single nucleotide polymorphisms (SNPs) only for these replicon plasmids (since they were the most associated with ARGs, see results) was created by aligning the plasmid sequences (hybrid assemblies for those subjected to ONT sequencing and short read-only assemblies obtained by MOB-suite for the remaining ones) to a hybrid reference. This hybrid reference was generated using the online MAFFT sequence alignment server (97) for three full plasmid sequences (hybrid assembly) from three of four different IncX1 putative groups, and then applying the NormalizeFasta function from GATK (98) and the ConsensusSequence function from *msa* package in R (88).

### Genomic analysis

The genetic relationship between isolates (*n* = 388) was assessed using a SNP-based approach. SNPs were identified by aligning the trimmed reads from the isolates to the reference *S*. Enteritidis genome P125109 (NC_011294.1) using Burrows-Wheeler Aligner (BWA) and SAMtools (99). The variant calling and consensus sequences were generated using BCFtools (100), whereas masking previously identified prophage regions, which were detected in advance in the reference sequence using PHASTER. The consensus sequences were concatenated to create a multi-FASTA alignment and Gubbins (101) was used to filter out putative recombinant regions (highly polymorphic sites). The SNPs remaining were identified and extracted using snp-sites (102), and a maximum-likelihood phylogenetic tree was then built in RAxML (103) using the CAT model of rate heterogeneity (GTRCAT) and 10,000 rapid bootstrap replicates. The tree was rooted using a sequence from *S*. Paratyphi A (GenBank accession number SRR3033248) as an outgroup.

The hierBAPS (hierarchical Bayesian Analysis of Population Structure) algorithm (104–107) was implemented through the “RhierBAPs” package (108, 109) in R to identify clusters in the sequenced population. BAPs level 1 clusters identified in the SNPs-based approach were considered as the main clades in the description of the results, whereas level 2 clusters were considered as subclades. Trees were visualized using iTols (110). In addition, hierarchical single linkage clustering of pairwise SNP distances was performed on the SNPs distance matrix at various distance thresholds (250, 100, 50, 25, 10, 5, and 0) with the *cutree* function from “stats” package. The generated code, defined as “SNP address” (27), allowed us to identify clusters of isolates at each level of the hierarchy. Isolates belonging to the same 5-SNPs clusters were considered potentially epidemiologically related as previously suggested for *S*. Enteritidis (24, 111).

In order to identify genomic differences between isolates in the different BAPs clades, the pangenome of the 388 isolates included in the phylogeny was reconstructed using the annotations from Prokka through Roary (112). Outputs generated by Roary included a gene presence/absence matrix, concatenated gene alignments, and a list of gene clusters. The association between the presence of specific genes/gene clusters and the BAPs clades was evaluated using Scoary (113), leading to the identification of genes significantly associated with a clade along with the p-values adjusted for multiple testing. Genes significantly associated with a clade were subjected to functional annotation using EggNOG-mapper (114, 115), which assigns functional roles to the genes identified based on the information provided by multiple databases including COG, KEGG, and Pfam. To confirm the results from the Roary/Scoary analysis in the case of the *dgcJ* gene (see results), BLAST was used to determine the presence and location of the full sequence of this gene (NZ_CP009091.1:1865142–1866635) in all the assemblies in this study. SAMtools with the “faidx” option were used to extract the gene region plus approximately 1,000 bp upstream and downstream from the assembled genomes. Then, a multi-alignment file of these sequences along with the downloaded complete gene was created to identify the differences between isolates.

Finally, the presence of the recently described SNP at position 25 in the promoter of the *mgrB* gene linked to colistin resistance (21) was determined as follows: the genomic region spanning positions 1,939,790 to 1,939,720 (approximately 70 bp) of the reference sequence of *S*. bongori N268-08 (NC_021870.1) was downloaded to obtain the entire *mgrB* gene region, including the *PhoP* promoter. Consensus sequences of the 388 isolates included in the phylogeny corresponding to the genomic region 1,278,489–1,278,861 (~140 bp), marking the boundary between the end of the previous CDS annotation and the beginning of the next, were extracted. The reference sequence and consensus files were indexed, and the specific region (c:1,278,489–1,278,861; ~140 bp) was extracted for alignment. The downloaded region from NC_021870.1 was aligned with the extracted sequences and used to determine the annotation positions. Multiple sequence alignment was carried out using the MAFFT tool after the concatenation of the sequences of the extracted regions from the reference and each consensus sequence. The alignments were visualized and the genotypes (presence/absence of the SNP) were determined considering the BAPs clade and colistin-resistance phenotype when available.

## Data Availability

Illumina sequencing reads generated in this study have been deposited in ENA under projects PRJEB58304, PRJEB73549, and PRJEB49008 (Data Set S1). Nanopore reads generated in this study have been deposited in ENA under project PRJEB58304.

## SUPPLEMENTAL MATERIAL

Data Set S1 aac.00738-24-s0001.xlsx Metadata of the 298 study isolates and the 88 selected external isolates analyzed.

Text S1 aac.00738-24-s0002.docx Bacterial DNA extraction protocols used, and captions for all supplemental material.

Fig. S1 aac.00738-24-s0003.jpg Number of isolates analyzed per year and host.

Fig. S2 aac.00738-24-s0004.png Phylogenetic tree including only isolates in the 2011-2019 period.

Fig. S3 aac.00738-24-s0005.png SNP-based phylogeny showing SNP addresses.

Fig. S4 aac.00738-24-s0006.png Virulence factors and SPIs displayed on the phylogenetic tree.

Fig. S5 aac.00738-24-s0007.png Number of isolates analyzed per region and host.

Fig. S6 aac.00738-24-s0008.jpg Number of human isolates analyzed per origin (project) and year.

Fig. S7 aac.00738-24-s0009.jpg Proportion of isolates displaying each resistance phenotype per host.

Supplemental tables aac.00738-24-s0010.xlsx Tables S1 to S6.

ASM does not own the copyrights to Supplemental Material that may be linked to, or accessed through, an article. The authors have granted ASM a non-exclusive, world-wide license to publish the Supplemental Material files. Please contact the corresponding author directly for reuse.
