# Supplementary material for: Molecular epidemiology of Salmonella Enteritidis in humans and animals in Spain
Source: Antimicrob Agents Chemother. 2025 Mar 3;69(4):e00738-24. doi: 10.1128/aac.00738-24 (PMC11963599; doi:10.1128/aac.00738-24)
Supplement: Text S1 — Bacterial DNA extraction protocols used, and captions for all supplemental material. [file aac.00738-24-s0002.docx]

**Bacterial DNA extraction:**

**Illunina sequencing**

A set of 176 (VISAVET) were sequenced in a Illumina MiSeq platform using v3 reagents with 2x300 cycles. DNA was extracted and purified using commercial kits (Hernández et al. 2017) and paired-end libraries were prepared using Nextera XT kit by the standard protocols (Illumina 2018).

The remaining 68 isolates, coming from human clinical cases (PI14CIII/00051, PI17CIII/00024, outbreaks and surveillance), were sequenced in an Illumina NextSeq platform. DNA was extracted using NZY Tissue gDNA isolation kit (Nzytech, Portugal) and paired-end libraries were prepared using Nextera DNA Flex Library preparation kit (Ilumina Inc.) for multiplexed sequencing.

**ONT sequencing**

A set of thirteen isolates (see in results), representing a diversity of plasmid replicons of interest containing ARGs, were selected for long-read sequencing on an Mk1C MinION device (Oxford Nanopore Technologies (ONT), UK). All isolates were cultured overnight in BHI broth (BD) at 37°C and pelleted.

Nine of the thirteen isolates (S1 dataset) were incubated in DNA/RNA shield (Zymo Research) at 7°C until DNA extraction (1-2 days later) was performed by semi-automated extraction using the MagCore Genomic Bacterial DNA Kit™ with a 60 μL elution volume (Atrida, Amersfoort, The Netherlands), following the manufacturer’s instructions. DNA of the remaining four strains (Supplementary File 1) was extracted from axenic cultures with the Qiagen DNA blood & tissue kit (QIAGEN) following the manufacturer’s instructions.

In both cases the long-read libraries were prepared using the Rapid Barcoding Sequencing kit (SQK-RBK004), loaded onto an R9.4.1 MinION flow cell (FLO-MIN106) and sequenced for 72h on a MinION Mk1C device. Basecalling and demultiplexing were carried out with Guppy (v6) and the MinKNOW control software was used to acquire raw signal from the device and raw reads (.fastq or .fast5 files).

**Captions of supplementary materials**

**Data Set S1.** Metadata of the 298 study isolates and the 88 selected external isolates analyzed.

**Text S1** Bacterial DNA extraction protocols

**Fig. S1.** Number of isolates analyzed per year and host.

**Fig. S2.** Phylogenetic analysis including only isolates in the 2011-2019 period

**Fig. S3.** SNP-based phylogeny showing SNP addresses.

**Fig. S4.** Virulence factors and SPIs displayed on the phylogenetic tree

**Fig. S5**. Number of isolates analyzed per region and host

**Fig. S6.** Number of human isolates analyzed per origin (project) and year)

**Fig. S7.** Proportion of isolates displaying each resistance phenotype per host.

**Supplementary tables**

**S1 Table.** Antimicrobial resistance-conferring genes/mutations found among the isolate collection.

**S2 Table.** Characteristics of the plasmids identified in the sequenced strains.

**S3 Table**. Results of the Roary/Scoary analysis

**S4 Table**. ECOFFs and EUCAST/CLSI clinical breakpoints available for the antimicrobials used in the phenotypic characterization.

**S5 Table**. Bioinformatic tools and parameters used in the pipeline applied in the study.

**S6 Table**. Location (chromosomal or plasmid) of plasmid replicons and antimicrobial resistance genes found on hybrid assemblies.
