# Supplementary figures and images for: Molecular epidemiology of Salmonella Enteritidis in humans and animals in Spain
Source: Antimicrob Agents Chemother. 2025 Mar 3;69(4):e00738-24. doi: 10.1128/aac.00738-24 (PMC11963599; doi:10.1128/aac.00738-24)

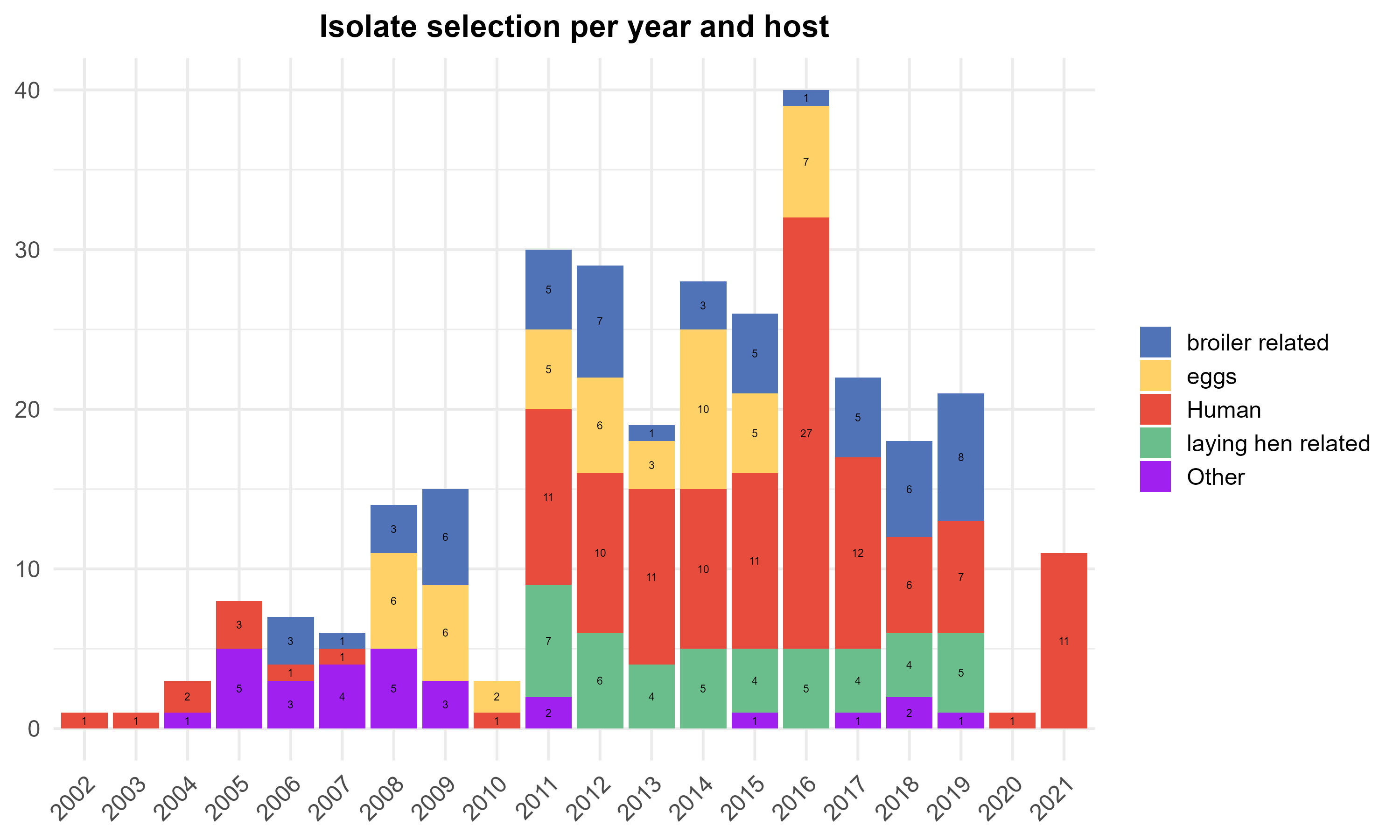

Supplement: Fig. S1 — Number of isolates analyzed per year and host. [file aac.00738-24-s0003.jpg]

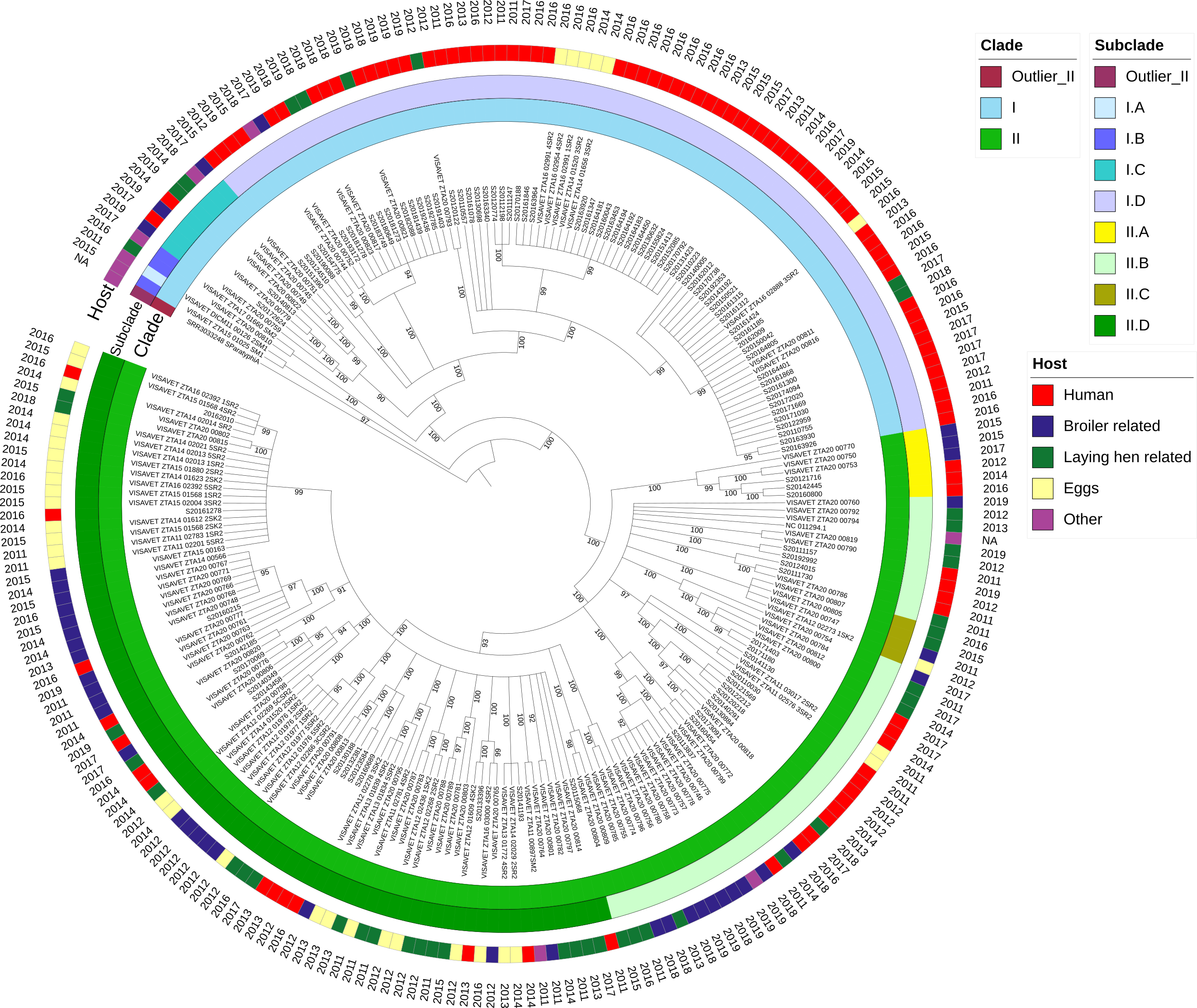

Supplement: Fig. S2 — Phylogenetic tree including only isolates in the 2011-2019 period. [file aac.00738-24-s0004.png]

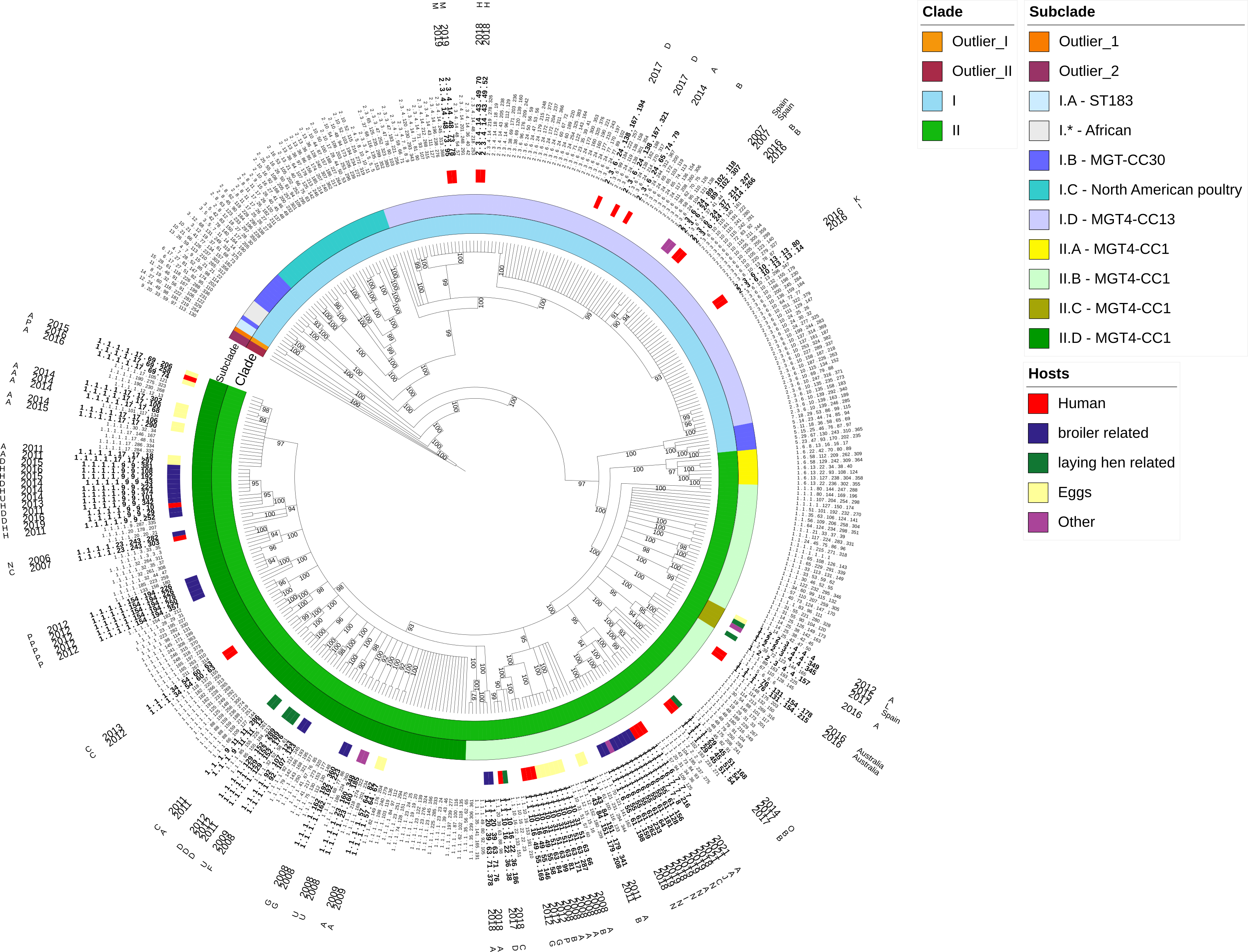

Supplement: Fig. S3 — SNP-based phylogeny showing SNP addresses. [file aac.00738-24-s0005.png]

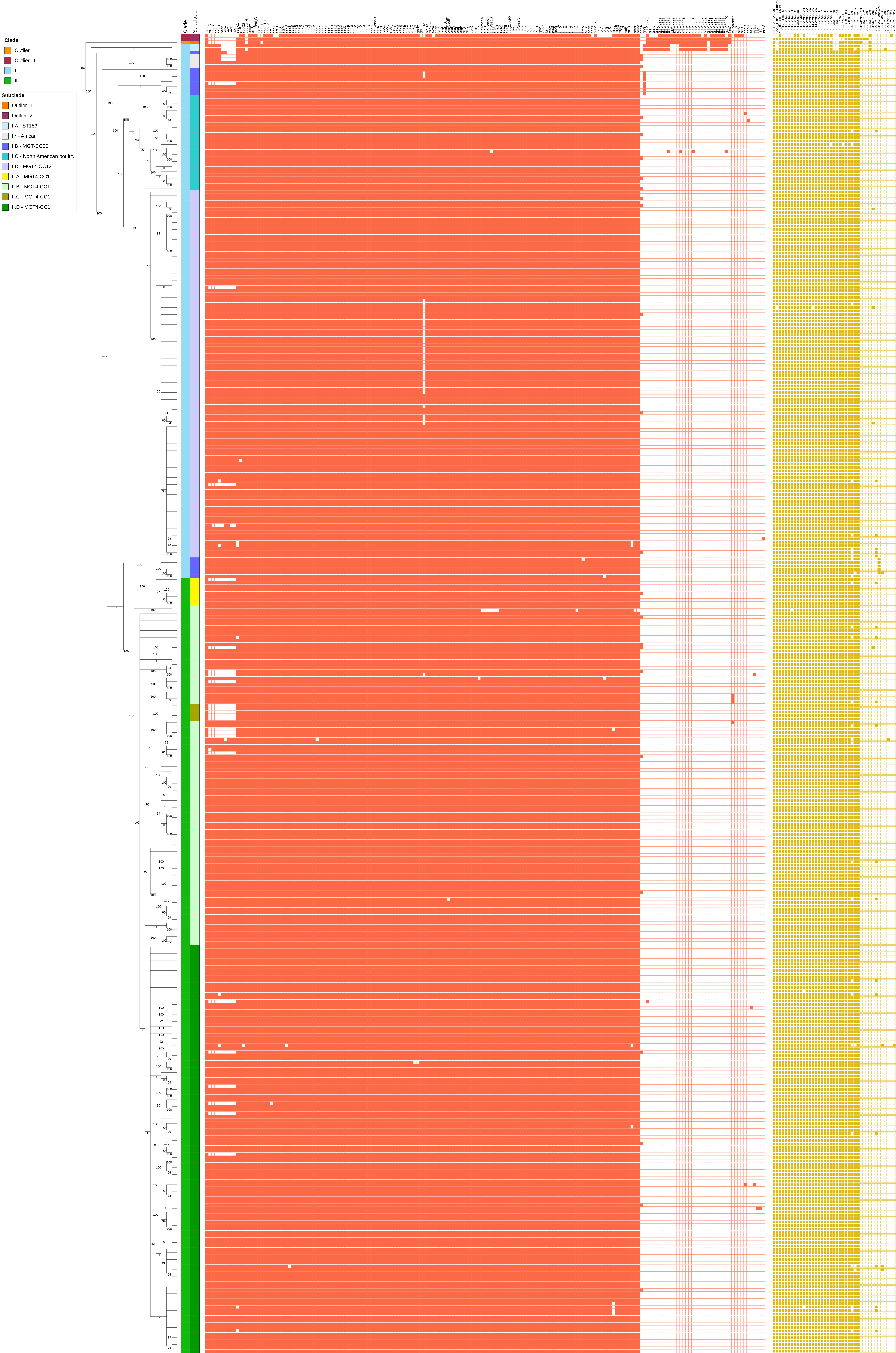

Supplement: Fig. S4 — Virulence factors and SPIs displayed on the phylogenetic tree. [file aac.00738-24-s0006.png]

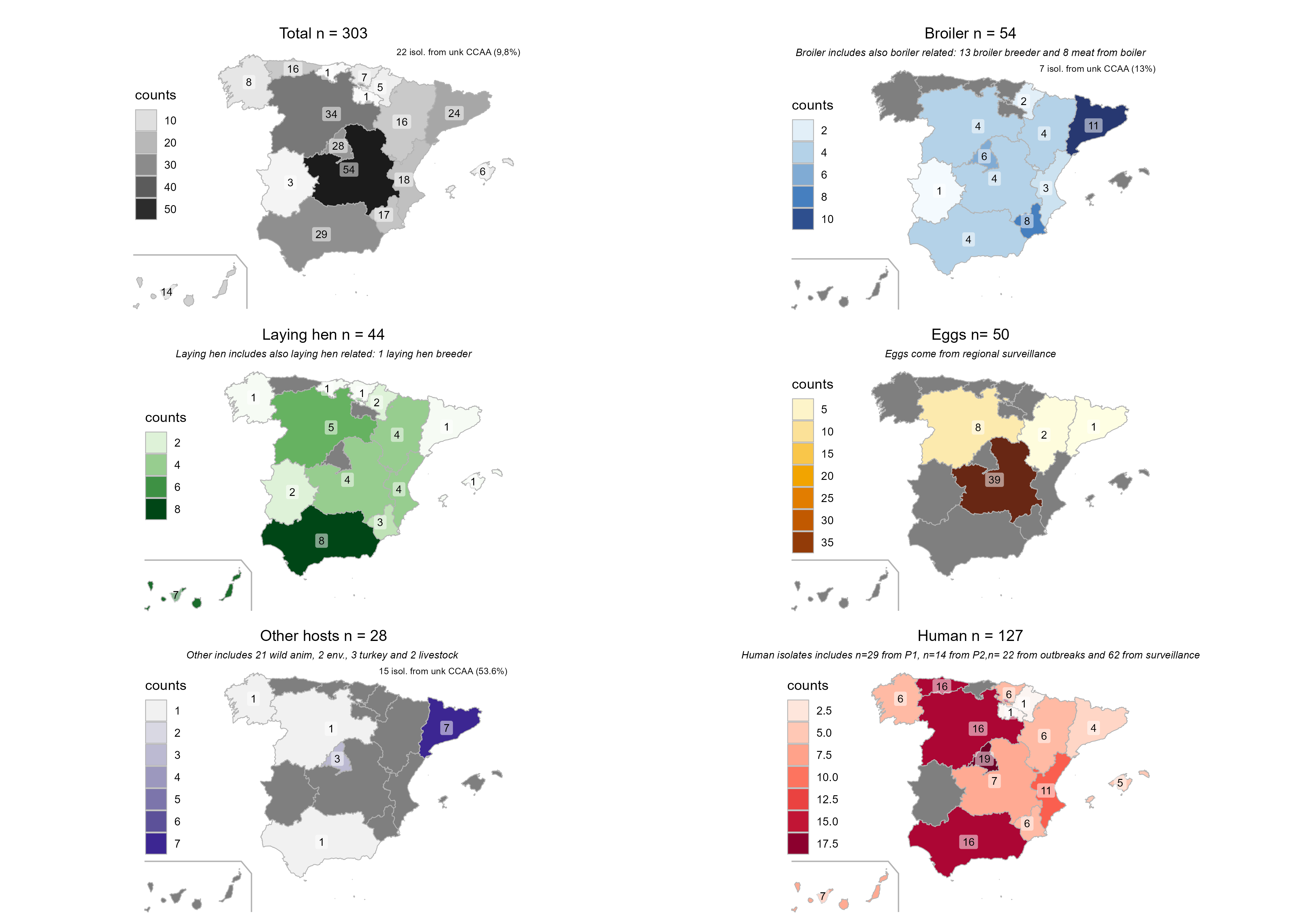

Supplement: Fig. S5 — Number of isolates analyzed per region and host. [file aac.00738-24-s0007.png]

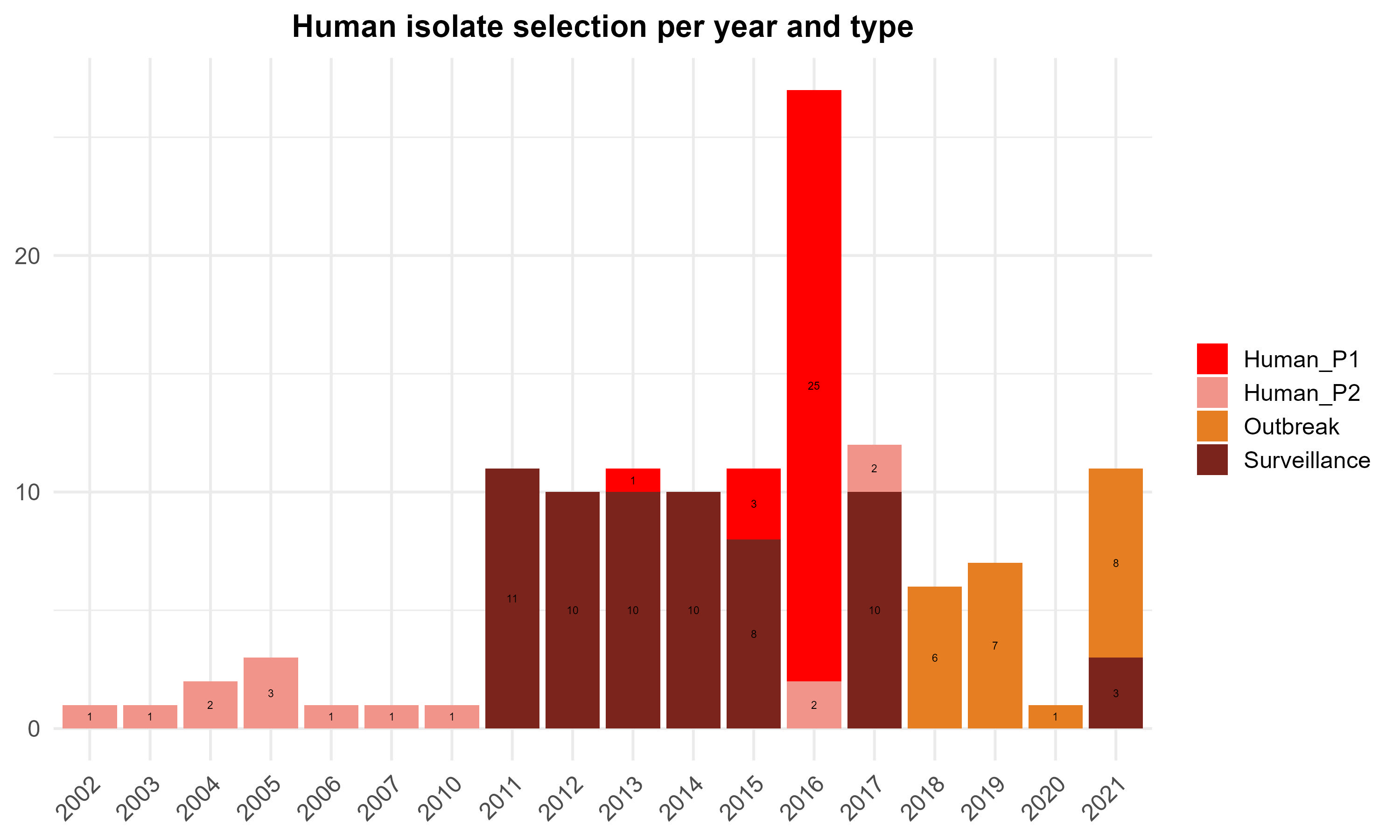

Supplement: Fig. S6 — Number of human isolates analyzed per origin (project) and year. [file aac.00738-24-s0008.jpg]

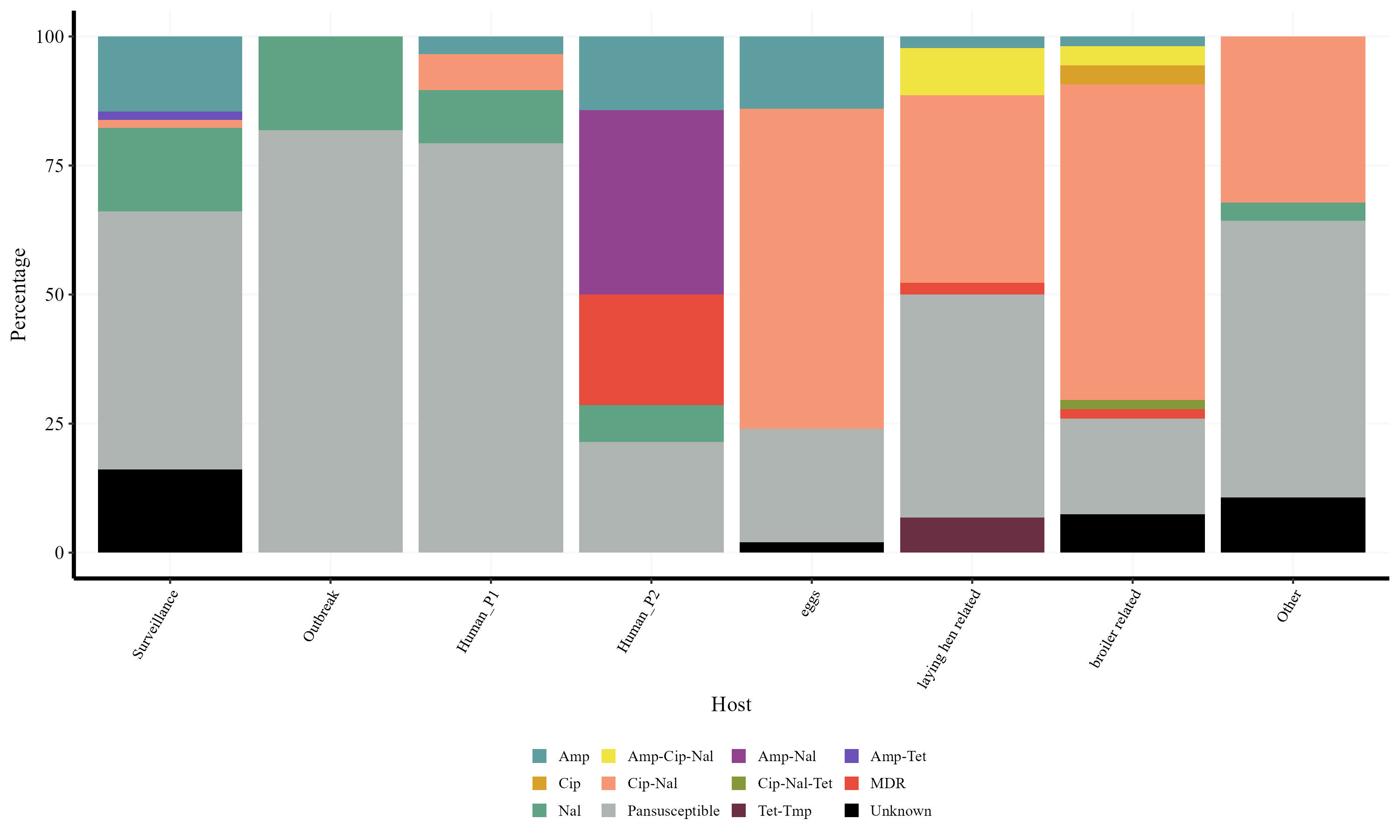

Supplement: Fig. S7 — Proportion of isolates displaying each resistance phenotype per host. [file aac.00738-24-s0009.jpg]
